# Epigenetic Repression of *p16^INK4A^* by Latent Epstein-Barr Virus Requires the Interaction of EBNA3A and EBNA3C with CtBP

**DOI:** 10.1371/journal.ppat.1000951

**Published:** 2010-06-10

**Authors:** Lenka Skalska, Robert E. White, Melanie Franz, Michaela Ruhmann, Martin J. Allday

**Affiliations:** Section of Virology, Division of Infectious Diseases, Faculty of Medicine, Imperial College London, London, United Kingdom; Emory University, United States of America

## Abstract

As an inhibitor of cyclin-dependent kinases, p16^INK4A^ is an important tumour suppressor and inducer of cellular senescence that is often inactivated during the development of cancer by promoter DNA methylation. Using newly established lymphoblastoid cell lines (LCLs) expressing a conditional EBNA3C from recombinant EBV, we demonstrate that EBNA3C inactivation initiates chromatin remodelling that resets the epigenetic status of *p16^INK4A^* to permit transcriptional activation: the polycomb-associated repressive H3K27me3 histone modification is substantially reduced, while the activation-related mark H3K4me3 is modestly increased. Activation of EBNA3C reverses the distribution of these epigenetic marks, represses *p16^INK4A^* transcription and allows proliferation. LCLs lacking EBNA3A express relatively high levels of p16^INK4A^ and have a similar pattern of histone modifications on *p16^INK4A^* as produced by the inactivation of EBNA3C. Since binding to the co-repressor of transcription CtBP has been linked to the oncogenic activity of EBNA3A and EBNA3C, we established LCLs with recombinant viruses encoding EBNA3A- and/or EBNA3C-mutants that no longer bind CtBP. These novel LCLs have revealed that the chromatin remodelling and epigenetic repression of *p16^INK4A^* requires the interaction of both EBNA3A and EBNA3C with CtBP. The repression of *p16^INK4A^* by latent EBV will not only overcome senescence in infected B cells, but may also pave the way for *p16^INK4A^* DNA methylation during B cell lymphomagenesis.

## Author Summary

We previously showed that two Epstein-Barr virus latency-associated proteins—EBNA3A and EBNA3C—contribute to enhanced B cell survival by inhibiting the expression of the death-inducing protein BIM. This repression involves remodelling of the *BIM* gene promoter by polycomb proteins and DNA methylation within an unusually large CpG-island that flanks the transcription initiation site. Here we show that the same two proteins, EBNA3A and EBNA3C, functionally cooperate in the polycomb-mediated chromatin remodelling of another tumour suppressor gene, *p16^INK4A^*, that encodes a cyclin-dependent kinase inhibitor capable of blocking cell proliferation. Both EBV proteins can bind the highly conserved co-repressor of transcription CtBP, and these interactions appear to be required for the efficient repression of *p16^INK4A^*. Thus by utilising the polycomb system to induce the heritable repression of two major tumour suppressor genes—one that induces cell death (*BIM*) and one that induces growth arrest (*p16^INK4A^*)—EBV profoundly alters latently infected B cells and their progeny, making them significantly more prone to malignant transformation.

## Introduction

*In vitro*, EBV can very efficiently induce the activation and continuous proliferation of resting human B lymphocytes. The resulting lymphoblastoid cell lines (LCLs) carry the viral genome as extra-chromosomal episomes and express only nine ‘latent’ EBV proteins. There are six nuclear antigens (EBNAs 1, 2, 3A, 3B, 3C & LP), three membrane associated proteins (LMP1, LMP2A & 2B) and in addition several untranslated RNA species. Together these factors activate the quiescent B cells and sustain their proliferation while maintaining the viral episome in its extra-chromosomal state (reviewed in [1]). Current data on the persistence of EBV in humans are consistent with the viral genome residing long-term in the resting memory B cell compartment. This occurs in at least of 90% of the world's population. However, to establish persistence EBV generally infects resting (naïve) B cells in Waldeyer's ring of the oropharynx and drives these to proliferate as activated B-blasts. This transient expansion of an infected B-blast population is generally accompanied by migration into germinal centres and differentiation to become centroblasts and centrocytes and finally resting memory B cells. The precise series of events that the EBV-positive B cells undergo to reach the memory compartment is unknown, but it appears to involve the regulated, sequential silencing of EBV genes encoding latency-associated proteins [2], [3].

Although infection is generally asymptomatic, EBV can be the causative agent in the benign self-limiting lymphoproliferation, infectious mononucleosis (IM). Uncontrolled proliferation of latently infected B cells in the immunocompromised may result in a chronic form of IM, a chronic polyclonal B-lymphoproliferative disorder called post-transplant lymphoproliferative disease (PTLD) or rarely the development of malignant lymphoma. Individuals co-infected with malaria (mainly children) or HIV (mainly adults) may be at increased risk of developing EBV-associated B cell lymphomas, including Burkitt's lymphoma (BL) and diffuse large B cell lymphoma [4].

EBNA3A, EBNA3B and EBNA3C are considered to comprise a family which probably arose in primate gamma-herpesvirus evolution by a series of gene duplication events since they have the same gene structure (ie a short 5′ coding exon and a long 3′ coding exon), are arranged in tandem in the EBV genome and share limited but significant amino acid sequence homology. EBNA3 transcripts are alternatively spliced from very long mRNAs generally initiated at the Cp latency promoter and LCLs have only a few copies of these transcripts per cell, suggesting their expression is very tightly regulated and the turnover of the EBNA3s is slow [1], [5]. Although they are related and the three proteins have limited homology, there is nothing to suggest that they have extensively redundant functions. Genetic studies using recombinant viruses originally indicated that EBNA3A and EBNA3C are essential for the efficient *in vitro* transformation or immortalisation of B cells, whereas EBNA3B is dispensable [6], [7]. However, under the appropriate conditions, with feeder cells present, it has been possible to establish EBNA3A-negative LCLs ([8]; our unpublished data).

Each EBNA3 protein binds to the cellular DNA-binding factor RBP-JK (also known as CBF1). This is the same protein that binds to, and targets to DNA, the EBV transactivator EBNA2 and the NOTCH-IC effector of the NOTCH signalling pathway. EBNA3A, EBNA3B and EBNA3C can repress Cp reporter plasmids and plasmids containing multiple RBP-JK/CBF1 binding sites derived from Cp ([9], [10], [11], [12]; P. Young and MJA, unpublished data). Since Cp is generally the promoter for all EBNA mRNA initiation in LCL cells, the EBNA3 proteins probably contribute to a negative auto-regulatory loop. In addition all three EBNA3s exhibit robust repressor activity when targeted directly to DNA by fusion with the DNA-binding domain of Gal4 and they all interact with one or more cellular factor(s) involved in transcriptional repression or silencing; these include histone deacetylases (HDACs) and CtBP ([11], [12], [13], [14], [15], [16], [17]; P. Young and MJA, unpublished data).

CtBP (C-terminal binding protein) was initially discovered as a cellular factor interacting with the C-terminus of adenovirus E1A oncoprotein and subsequently identified as one of a highly conserved family of co-repressors of transcription (reviewed in [18]). Most of the factors that bind to CtBP and negatively regulate transcription contain at least one conserved Pro-Leu-Asp-Leu-Ser (“PLDLS”) CtBP-interaction domain (or close variant) that is necessary and probably sufficient for the interaction. CtBP-containing complexes can coordinate biochemical and enzymatic events that convert transcriptionally active chromatin directly to a repressive or silent state ([19], [20]). Moreover there is also good evidence that CtBP is involved in the regulation of cell proliferation since it has been shown that CtBP forms a link between human polycomb group (PcG) proteins and pRb [21] and that CtBP and PcG complexes both regulate elements in the *CDKN2A* locus [22], [23].

EBNA3A and EBNA3C each bind to CtBP *in vitro* and *in vivo* but this binding correlates only partially with their ability to repress transcription when targeted to DNA in transient reporter assays. However, the interaction correlates extremely well with their ability to behave as cooperating nuclear oncogenes when introduced into primary rodent fibroblasts with oncogenic Ha-*RAS* [16], [17], [24]. Since in this type of assay, the oncogene Ha-*RAS* alone triggers exit from the cell cycle and premature senescence via the induction of the p16^INK4A^ and/or p19^ARF^ proteins encoded by the *CDKN2A* locus [23], [25], this suggests that EBNA3A and EBNA3C can each rescue primary fibroblasts from growth arrest and senescence.

Further evidence that EBNA3C deregulates the cell cycle came with the demonstration that when over-expressed it overcomes a mitotic metaphase checkpoint and induces polyploidy and multi-nucleation, eventually leading to cell death [26]. However, the molecular details of its action in mitosis have remained elusive. A reported interaction with CHK2 suggests that it could have a role in the transition from G2 to mitosis, but this has not yet been proven [27] and although it has been reported that EBNA3C may repress the transcription of the mitotic regulator BUBR1 in one B cell line, this has not been extended to LCLs [28]. Several recent reports indicate that EBNA3C can also directly associate with multiple other factors involved in the regulation of cell cycle progression and/or the G1/S checkpoint. These include Cyclin A; the ubiquitin ligase SCF^SKP2^; the tumour suppressor pRb; the oncoprotein MYC; MDM2 in a complex with p53 and p53 alone [29], [30], [31], [32], [33], [34]. It remains to be determined if these interactions occur in infected B cells and whether they are functionally significant.

The most direct and compelling evidence that EBNA3C modulates the cell cycle during EBV-mediated transformation of B cells into LCLs comes from Maruo and colleagues. Using a recombinant Akata EBV made conditional for EBNA3C function by fusing EBNA3C with a modified oestrogen receptor, they revealed that EBNA3C represses expression of the cyclin-dependent kinase inhibitor p16^INK4A^ in LCLs. Removing the inducer of EBNA3C activity (4-hydroxytamoxifen) from the culture medium results in an accumulation of both p16^INK4A^ mRNA and protein and in reduced cell proliferation [35].

EBNA3A also cooperates with Ha-RAS in the transformation and immortalization of REFs and there is again a remarkably good correlation between EBNA3A binding to CtBP and its ability to cooperate with oncogenic *ras* [17]. Furthermore, a 4-hydroxytamoxifen-dependent LCL conditional for EBNA3A function showed that in the absence of EBNA3A, cell proliferation gradually declines. Although it was not indicated whether this involved regulation of the *CDKN2A* locus [36], a more recent report of a microarray screen has indicated that repression of *p16^INK4A^* transcription in LCLs is associated with EBNA3A expression [8].

We recently demonstrated that EBV represses transcription of the gene encoding the pro-apoptotic BCL-2-related family member BIM [37], [38]. The repression of *BIM* transcription initially involves a polycomb repressive complex, PRC2, where the histone methyltransferase EZH2, which together with SUZ12 and EED, is responsible for establishing the epigenetic modification H3K27me3 (tri-methylation of lysine 27 on histone H3) (reviewed in [39], [40]). H3K27 methylation of *BIM* may then be followed by DNA methylation of sites within the CpG-island flanking the *BIM* transcriptional initiation site ([38] and our unpublished data). Since EBNA3A and EBNA3C are necessary for the chromatin remodelling and epigenetic repression of *BIM*, here we have examined the *p16^INK4A^* locus and the roles EBNA3A and EBNA3C play in regulating its epigenetic status.

## Materials and Methods

### Generation of recombinant EBV-BACs

An EBNA3C-HT fusion protein (3CHT) in the B95-8 background was constructed with the same linking sequence and 4-hydroxytamoxifen-sensitive murine estrogen receptor that has previously been described in the Akata background [35]. The connection between the 3C and HT is a single proline residue between the last amino acid of EBNA3C and amino acid 281 of the murine estrogen receptor alpha (modified by G525R to make it 4-hydroxytamoxifen-specific. This fusion was recombined into the B95-8 bacterial artificial chromosome (BAC) [41] using previously described methods [37], [42] to produce two independent BACs containing 3CHT (A and C).

A set of CtBP-binding-mutant viruses were generated in which the EBNA3A and/or EBNA3C binding site(s) for CtBP were replaced with previously characterised mutations that lack the ability to bind CtBP [16], [17]. This was achieved by a sequential set of recombinations, initially mutating the pair of CtBP binding sites in EBNA3A (to generate the 3A^CtBP^ mutant). The EBNA3C binding site for CtBP in this was then mutated to create a virus genome lacking all CtBP binding sites among the EBNA3s (E3^CtBP^). Then the EBNA3A mutant sequence was replaced with wild-type sequence, leaving only the EBNA3C sequence as mutant (3C^CtBP^) and finally the EBNA3C sequence was reverted to wild-type sequence, generating the CtBP revertant (rev^CtBP^).

### Cell culture

Established LCLs were cultured in RPMI-1640 medium (Invitrogen) supplemented with 10% fetal calf serum, penicillin and streptomycin. LCL 3CHT were cultured with addition of 400nM of 4-hydroxytamoxifen (HT, Sigma). After the infection of primary B cells, LCLs were grown to a volume and density suitable for freezing multiple aliquots (typically about 60ml at a density of 3×10^5^ cells/ml or greater). This took 4–8 weeks for WT-EBV, revertant and 3CHT LCLs and 6–12 weeks for the EBNA3A and CtBP mutant LCLs.

Cells recovered from liquid nitrogen were cultured for about 10 days (with HT if necessary) before the start of any experiment. At the end of an experiment the cells were discarded. Twenty-four hours before any experimental treatment, cells were seeded at a density of 2.5×10^5^ cells/ml.

### Infection of 1° B cells with recombinant EBV

Virus was produced by transfection of recombinant BACs into HEK293 cells [(ATCC, CRL-1573), a kind gift of Claire Shannon-Lowe, University of Birmingham] and selection of clonal Hygromycin B-resistant cell lines, which were screened for integrity of EBV genome by episome rescue and pulsed-field gel analysis of BAC restriction digests (not shown). Infectious virus was produced by the transfection of EBV-BAC-containing 293 cells with BZLF1 and BALF4 expression constructs [43], and after 4 days, supernatant was filtered through 0.45 µm filters. Virus titre was assessed by infection of Raji cells and counting green cells on an inverted fluorescent microscope after enhancement of GFP expression by overnight treatment with 5 nM TPA and 1.25 mM sodium butyrate. Virus titres were typically in the range of 50 to 250 raji green units (rgu) per microlitre of tissue culture supernatant.

B-cells for generation of LCL-3CHT-A and -C and EBNA3A-knockout LCLs (and for limiting dilution experiments – see below) were isolated from buffy coat residues (UK blood transfusion services) by centrifugation over ficoll. CtBP mutant LCLs and 3CHT-LCL B, D and E (described herein) were generated by infection of PBLs isolated from donated EBV-seronegative blood (a kind gift of Ingo Johannessen, University of Edinburgh). Three donors were used (D1, D2 and D3). 3CHT-LCL B, D and E were made by infection of in blood from donors 1, 3 and 2 respectively with EBV-3CHT-A virus. Essentially, between 50 µl and 1 ml of virus was added to 10^6^ PBLs (typically 2–8% of which are B-cells by FACS for CD20; not shown) in a well of a 24 well plate, and cultured initially in RPMI supplemented with 15% FCS, supplemented with Cyclosporine A (500 ng/ml) for the first 2–3 weeks. Once LCLs had grown out into large culture volumes, the FCS level in the medium was reduced to 10%.

### Limiting dilution and cell growth assays

Virus stocks were diluted to 2×10^4^ Raji green units (rgu) per ml in RPMI supplemented with 10% FCS, and ten-fold serial dilutions used to generate virus concentrations down to 0.2 rgu/ml. The virus was added to an equal volume of PBLs at 2×10^6^ cells per ml, and 1 ml was seeded per well in a 24 well plates. Two virus preparations from independent 293 cell producer lines were used for each virus mutant, and 6 wells for each virus concentration. 24 wells with no virus were used to control for spontaneous immortalisation of B-cells. After a week, the culture volume was increased to 2 ml and half the culture volume was replaced weekly thereafter. For the first 2 weeks, the medium was supplemented with cyclosporine A (500 ng/ml). Cell growth was monitored and wells were scored positive or negative after 40 days, based on the presence of clumps of cells characteristic of LCLs. To assess growth rate of CtBP-mutant LCLs, 5×10^4^ cells per ml of cells were seeded in 10 ml in a flask. 0.5 ml was removed every day and live cells (by trypan blue staining to exclude dead cells) were counted on a haemocytometer.

### Flow cytometry

To analyse cell cycle distribution, 2×10^6^ LCL cells were fixed in 80% ethanol, incubated in PI solution [PBS containing 18 µg/ml propidium iodide (PI) and 8 µg/ml RNase A (Sigma Aldrich)] at 4°C for 1 h before flow cytometric analysis. To quantify cells in S phase, cells were pulsed with 10 µM 5-bromo-2′-deoxyuridine (BrdU) (Sigma Aldrich) for 1 h at 37°C, harvested immediately after the pulse, fixed in 80% ethanol and co-stained with FITC-conjugated anti-BrdU mAb (Becton Dickinson) for 1 h at room temperature and PI solution for 1 h at 37°C.

### Western immunoblotting

Western blotting was performed essentially as described previously [16]. Briefly, proteins extracted using RIPA buffer, or in some cases whole cell lysates were resolved by sodium dodecyl sulphate–polyacrylamide gel electrophoresis (SDS–PAGE) and transferred to Protran nitrocellulose membranes (Schleicher and Schuell Bioscience, Dassel, Germany). Membranes were blocked with 5% milk powder in PBS/0.05% Tween 20, probed with appropriate primary and HRP-conjugated secondary antibodies. ECL kit (Amersham Biosciences, Chalfont St Giles, UK) was used for visualization. Following primary antibodies were used: mouse monoclonal anti-γ-tubulin (Sigma, T6557), sheep polyclonal anti-EBNA3A (Exalpha, USA), mouse monoclonal anti-EBNA3C (A10, kind gift from Prof. Martin Rowe, University of Birmingham), mouse monoclonal phospho-independent anti-Rb (BD Pharmingen, 554136), rabbit polyclonal anti-phospho-Rb (Ser 807–811) (Cell Signaling, 9308), mouse monoclonal anti-E2F1 (Millipore, mixed clones cKH20 and KH95, 05-379), mouse monoclonal anti-p16^INK4A^ (clone JC8, kind gift from Dr Gordon Peters, Cancer Research UK), rabbit polyclonal anti-p130 (Santa-Cruz, c-20, sc-317), rabbit polyclonal anti-p107 (Santa Cruz, c-18, sc-138), mouse monoclonal anti-EBNA2 (clone PE2, Dako), mouse monoclonal anti-LMP1 (clone CS1-4, Dako), mouse monoclonal anti-EBNA-LP (clone JF186, gift of Paul Farrell), rat monoclonal anti-LMP2A (clone 14B7, AbCam 59026), rabbit polyclonal anti-CtBP [16] and rat monoclonal anti-EBNA3B (clone 6C9, provided by Elizabeth Kremmer, Munich; Popp *et al,* submitted for publication).

### mRNA and Quantitative real time PCR (Q RT-PCR)

For Q RT-PCR, RNA was extracted from approximately 5×10^6^ cells for each cell line using the RNeasy mini kit from Qiagen and following the manufacturer's instructions. One microgram of each RNA sample was reverse-transcribed using SuperScript III First-Strand Synthesis Supermix for qRT-PCR (Invitrogen). Between 0.5–1% of cDNA product (equivalent to 5–10 ng RNA) was used per qPCR reaction. qPCR was performed on an ABI 7900HT real-time PCR machine using Platinum Sybr Green qPCR SuperMix UDG kit (Invitrogen). The cycling conditions were 95°C for 20 sec, followed by 40 cycles of 1 sec at 95°C, 20 sec at 60°C on a fast block. Dissociation curve analysis was performed during each run to confirm absence of non-specific products. Sequences of the assays used are listed in Table S1.

Standard curves, used to standardise amplification efficiency, were produced by six 5-fold serial dilutions of a mixture containing all cDNA samples used. Results were analyzed with qbase^PLUS^ software (Biogazelle, Ghent, Netherlands). Precise normalization was achieved using internal average control calculated from the controls (housekeeping genes – bold in Table S1) with highest stability rating (usually ALAS1 and GNB2L1). The calculated errors in the graphs are the standard errors from three replicate qPCR reactions for each mRNA.

### Chromatin immunoprecipitations (ChIP)

Chromatin immunoprecipitations for methylated histone H3 were performed essentially as described previously [38]. Precipitated DNA was cleaned using QIAquick Gel Extraction Kit (Qiagen) and was assayed by qPCR. Input DNA Ct was adjusted from the 5% used in the qPCR to 100% equivalent by subtracting 4.32 (Log2 of 20) cycles. ‘Percent input’ precipitated was then calculated by 100×2^∧^ (Ct adjusted input – Ct IP). Non-specific background was estimated by precipitation with IgG (data not shown since all values were below 0.03% of input). The error bars represent standard deviations from triplicate pPCR reactions for both input and IP. Sequences of the primers used in these assays are listed in Table S2.

## Results

### Inactivation of EBNA3C leads to proliferative arrest of LCL 3CHT that can be reversed by re-adding HT

In order to examine in detail the regulation of *p16^INK4A^* and cell proliferation by EBV, initially a recombinant EBV encoding a conditional EBNA3C was constructed. We employed the B95.8 EBV-BAC system used previously to generate EBNA3-knockout (KO) viruses and the design of fusion proteins described by Maruo and colleagues for fusing the carboxyl-terminus of EBNA3C to a modified estrogen receptor [35], [37]. EBNA3C fused to this estrogen receptor is dependent on 4-hydroxytamoxifen (HT) in the culture medium for its function and stability. Two independently generated viruses encoding the EBNA3C-HT-fusion (3CHT) were used to establish multiple lymphoblastoid cell lines (called LCL 3CHT).

**Figure 1:**
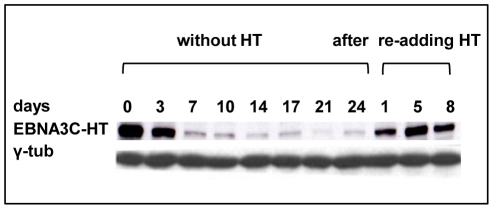
EBNA3C inactivation and reactivation in LCL 3CHT. Cells that had been grown in culture medium containing HT were washed and re-suspended in medium with HT omitted. After culturing for the number of days indicated, protein extracts were western blotted and probed with an anti-EBNA3C MAb to reveal EBNA3C-HT. After 17 days some cells were transferred to medium containing HT and after 1, 5 or 8 days samples were again taken for western blotting. The western blot was re-probed with anti-γ-tubulin to ensure equal loading of the proteins.

LCL 3CHT – with one notable exception described below – required HT in the culture medium for their proliferation. When HT was removed, after 3–7 days there was a dramatic reduction in the amount of EBNA3C detected by western blotting (for a representative example see Figure 1). This reduced expression is probably because the inactivated fusion binds to heat shock proteins and is targeted for proteasome-mediated degradation and is consistent with the behaviour of the equivalent fusion in the Akata strain EBV [35]. The LCLs were validated further by western blot analyses probing for each of the EBV latent proteins. As was previously reported in Akata, no consistent differences in steady state levels were seen. In the absence of HT a very slight increase in LMP1 was sometimes observed – this was also reported previously (Figure S1; [35] and data not shown).

**Figure 2:**
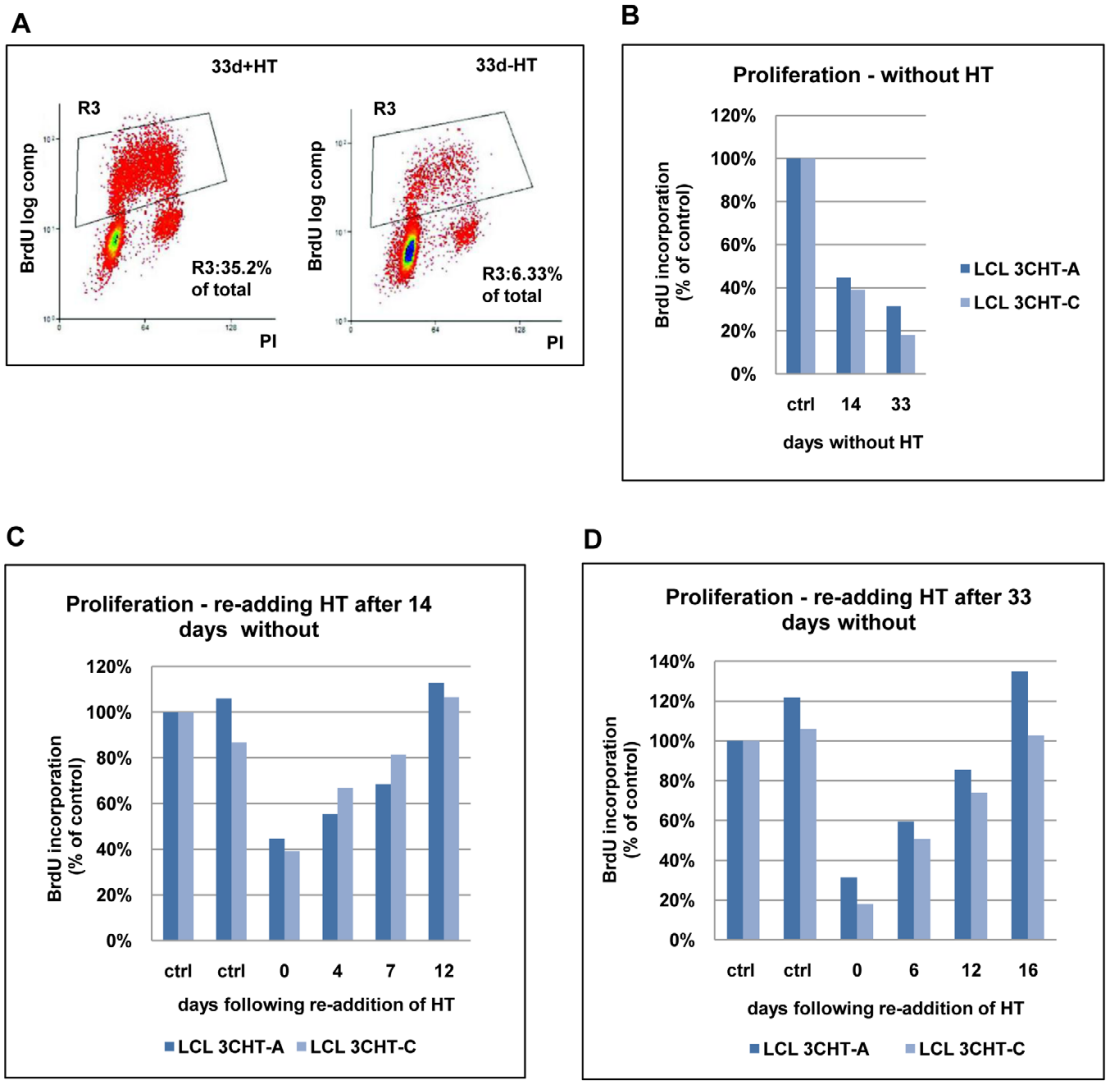
Proliferation of LCL 3CHT after inactivation and reactivation of EBNA3C. (**A**) LCL 3CHT cells were cultured either with HT (+HT) or for 33 days after the removal of HT (-HT) from the growth medium. Cells were pulsed for 1 hour with BrdU, harvested, fixed and stained with anti-BrdU-FITC and propidium iodide. The cells were analysed by flow cytometry. The gated BrdU-positive population is reduced in the absence of HT. (**B**) Two LCL 3CHT (-A and -C) generated from independent 3CHT-BACs using PBL from a single donor were analysed as in (A) after 14 and 33 days without HT. Histograms show BrdU incorporation relative to the control, cycling population. The control (ctrl) was a proliferating population of LCL 3CHT grown in medium with HT. (**C**) LCLs 3CHT-A and -C were cultured for 14 days in the absence of HT (0) and BrdU-incorporation was assayed as in (A). The incorporation of BrdU was also assayed as above after 4, 7 and 12 days after re-adding HT. (**D**) A similar experiment was performed on cells cultured without HT for 33 days.

In order to confirm that inactivation of EBNA3C compromises cellular DNA synthesis, BrdU incorporation was assessed. Two LCL 3CHT (-A & -C) cells were cultured with HT, then for 14 or 33 days after HT had been removed from the culture medium. Cells were then pulsed for 1 hour with BrdU, harvested, fixed and stained with anti-BrdU-FITC and propidium iodide. The cells were analysed by flow cytometry (see for example Figure 2A). In both LCL 3CHT, BrdU incorporation after 14 days without HT was reduced to about 40% of that in similar control cells cultured with HT and after 33 days the reduction in the proportion of cells entering S phase was even more profound (Figure 2B).

To establish whether the growth arrest could be reversed upon reactivation of EBNA3C-HT, HT was re-added after 14 or 33 days growth in its absence. Surprisingly, although the levels of EBNA3C-HT were re-established quite rapidly (within about 24 hours: see Figure 1), there was a significant delay before BrdU incorporation matched that of the controls. These cells clearly do not behave like a synchronised G1/S-arrested population released into S phase. When HT was re-added after 14 days without HT, it took 12 days to return to the level of proliferation seen in control cells (Figure 2C). When the HT was re-added after 33 days without HT, the period required to achieve full proliferation was extended to 16 days (Figure 2D). Since the restoration of cell proliferation after reactivation of EBNA3C is dependent on the time elapsed without functional EBNA3C, it is possible that a subpopulation of cells is being driven into in a state of irreversible arrest similar to senescence. These would be refractory to further pro-proliferative signals from EBNA3C and they would gradually accumulate. Alternatively there may be an intrinsic delay associated with the molecular processes necessary to re-induce cell cycle progression in individual arrested cells. It should be noted that, although viable, these non-proliferating cells did not stain positive for the operational mark of senescence β-galactosidase (data not shown).

### Proliferation of LCL 3CHT correlates with the regulation of p16^INK4A^ expression

Maruo and colleagues described how in the absence of functional EBNA3C, the cyclin-dependent kinase inhibitor p16^INK4A^ accumulates [35]. We needed to establish that similar changes in p16^INK4A^ levels occur in the B95.8-derived LCL 3CHT, and determine whether the response to EBNA3C reactivation correlated with (and could account for) the reduction in p16^INK4A^ expression and the changes in proliferation of LCL 3CHT described above.

**Figure 3:**
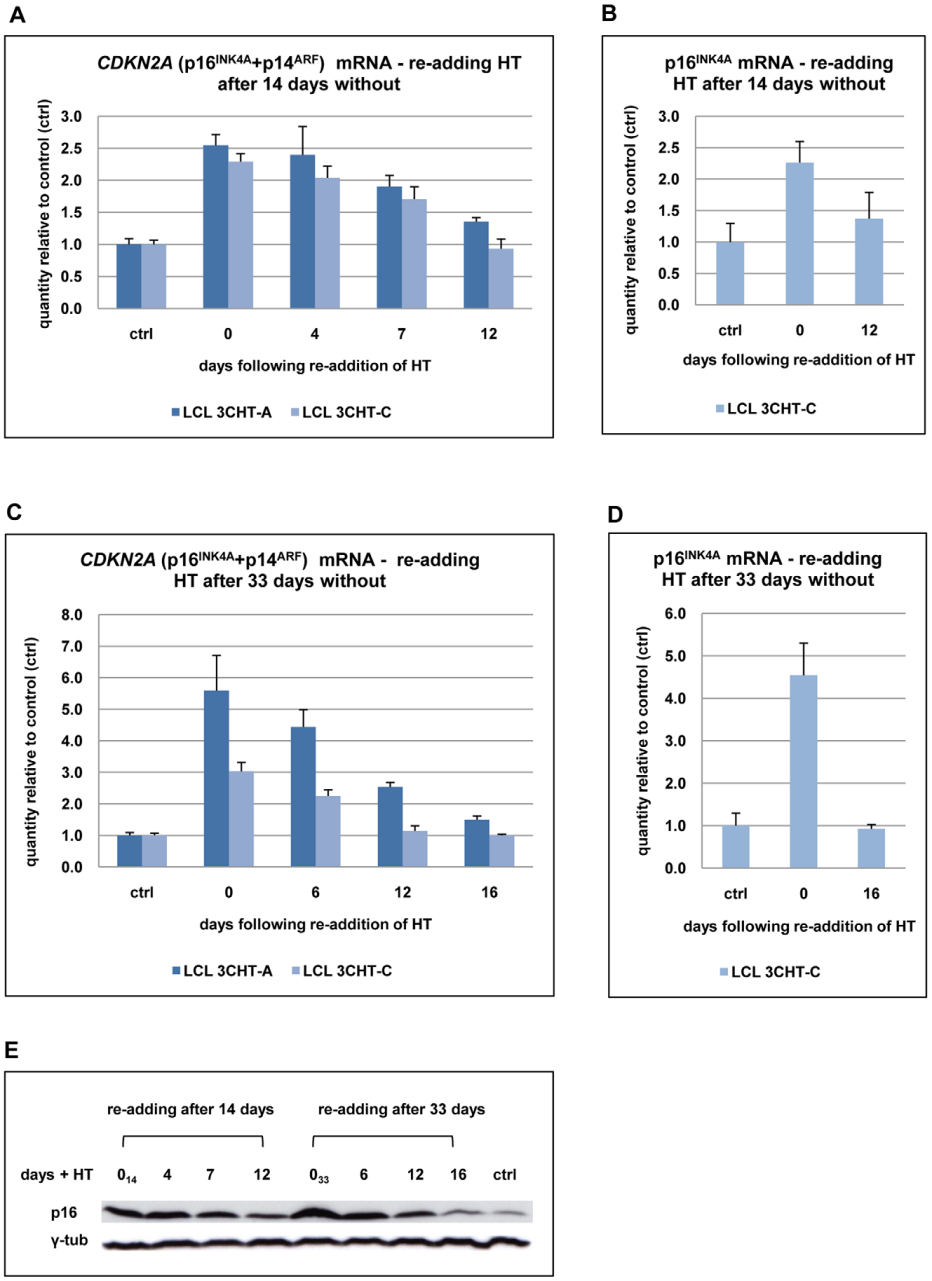
Repression of *p16^INK4A^* following reactivation of EBNA3C. (**A**) After 14 days without HT (0), total RNA was extracted from aliquots from two LCL 3CHT populations (-A and -C). HT was re-added to the remaining cells and further RNA samples were taken at the times indicated. Real time quantitative RT-PCR (qRT-PCR) was performed to quantify *CDKN2A* transcripts. The histogram corresponds to *CDKN2A* mRNA relative to that in control cycling populations of each LCL 3CHT. (**B**) As in (A) but using a *p16^INK4A^*-specific qPCR. (**C**) **&** (**D**) Similar assays to those described in (A) and (B) after 33 days without HT. (**E**) Western blots – probed with a p16^INK4A^-specific MAb – of protein extracts from LCL 3CHT-C cells to which HT was re-added after 14 or 33 days in its absence (0_14_ and 0_33_ respectively). The control (ctrl) is LCL 3CHT-C cells continuously cultured in medium with HT.

The DNA sequence of the unique first exon of *p16^INK4A^* is GC rich and is predicted to have extensive secondary structure under the conditions used for qPCR (based on DNA sequence analysis using Visual OMP). A quantitative RT-PCR assay specific for *p16^INK4A^* transcripts, which detects the amplicon located within the unique first exon of *p16^INK4A^* (Table S1), is quantitative only in the presence of large amounts of template. Therefore, a second assay that quantifies an amplicon located within the second and third exon shared by *p16^INK4A^* and *p14^ARF^* was designed (*CDKN2A* assay; Table S1), which is quantitative over a 5-log range of template concentration. These assays were used to show that the regulation of *p16^INK4A^* in B95.8-BAC LCL 3CHT is reversible (Figure 3). After 14 days in culture in the absence of HT, there was a 2-2.5-fold increase in *CDKN2A* transcripts relative to the control population. After re-adding HT into the medium, *CDKN2A* transcripts gradually decreased over the next 12 days (Figure 3A). This result was confirmed with the *p16^INK4A^*-specific assay (Figure 3B). Extending the period of culture in the absence of HT to 33 days resulted in a 3 to 5-fold increase in the level of *CDKN2A* transcripts. After re-adding HT to the medium, *CDKN2A* transcripts gradually decreased to the levels found in an actively proliferating culture, but it seemed to require at least 16 days (Figure 3C). This was also consistent with the results using the *p16^INK4A^*-specific primer set (Figure 3D).

Echoing the transcript data, p16^INK4A^ protein expression was gradually reduced after reactivation of EBNA3C in LCL 3CHT (Figure 3E). We conclude that regulation of p16^INK4A^ expression in LCL 3CHT undergoing changes in proliferation seems to be exclusively or at least predominantly at the level of transcription as has been previously shown in various types of cell, including LCLs [23], [35].

Taken together, the data confirm that p16^INK4A^ accumulates in LCL 3CHT cultured without HT and this inversely correlates with the proportion of cells entering S phase. The data show for the first time that when EBNA3C is reactivated by the re-addition of HT to the culture medium, the reverse occurs and the correlation between EBNA3C activity, *p16^INK4A^* transcription and proliferation holds true.

### Inactivation of EBNA3C leads to dephosphorylation of the retinoblastoma protein (Rb), reduced expression of p107 and an increase in p130; activation of EBNA3C reverses these processes

**Figure 4:**
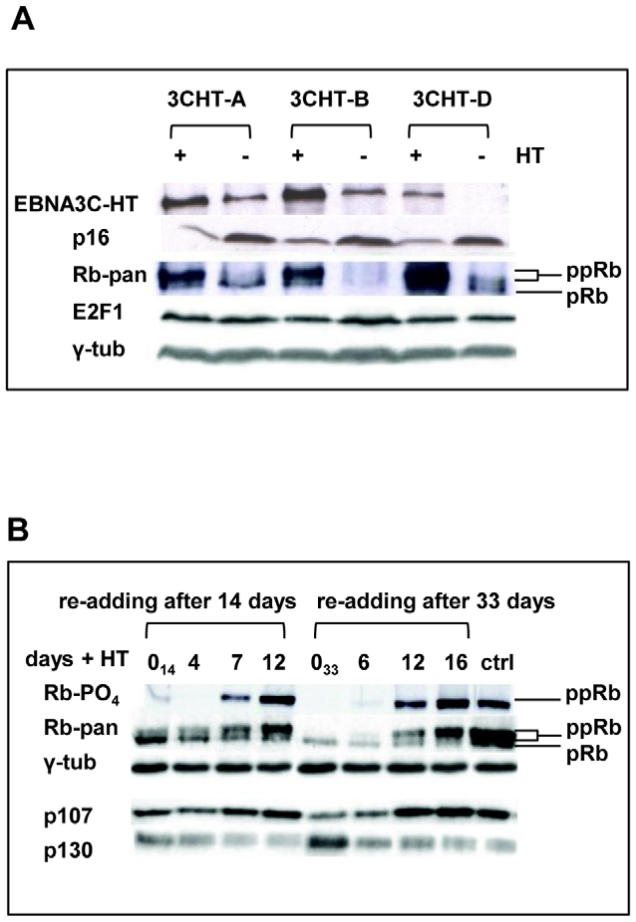
Inactivation of EBNA3C and accumulation of *p16^INK4A^* leads to de-phosphorylation of Rb, reduced expression of p107 and an increase in p130; activation of EBNA3C reverses these processes. (**A**) Western blot analyses of three LCL 3CHT lines performed on whole cell lysates from cells cultured with HT (+) without HT for 26 days (−). As the amount of EBNA3C protein reduces (and is inactivated) and p16^INK4A^ accumulates, so hyperphosphorylated Rb (ppRb) protein disappears and only hypophosphorylated Rb (pRb) is detected. The levels of E2F1 and γ-tubulin (γ-tub) remain unchanged irrespective of the culture conditions. (**B**) Western blot analysis of extracts from cells after re-addition of HT to cultures starved of HT for either 14 or 33 days (0_14_ and 0_33_ respectively). A pan-specific anti-Rb MAb and phospho-specific MAb (Ser 807–811) both show an increase in hyperphosphorylated Rb (ppRb) after HT was added. After 12–16 days the degree of phosphorylation is equivalent to that in the proliferating control LCL 3CHT population (ctrl). As Rb becomes phosphorylated, expression of p107 increases and expression of p130 decreases. The level of γ-tubulin (γ-tub) did not alter throughout the experiments.

In order to determine the consequences of p16^INK4A^ accumulation on the rest of the Rb-axis in the absence of functional EBNA3C, LCL 3CHT were cultured with HT (controls) and without HT for 14 and 33 days. Subsequently, HT was re-added on day 14 or on day 33. In arrested cells, Rb became hypophosphorylated (as revealed by both pan-specific and phospho-specific anti-Rb antibodies); as the arrest intensified after extended time without HT, reduced phosphorylation was accompanied by a slight reduction in the expression of Rb (Figures 4A and B). Simultaneously the Rb-related p130 protein accumulated and the amount of p107 was reduced (Figure 4B, annotated 0_14_ and 0_33_). This was particularly apparent after 33 days. Consistent with the data showing recovery after re-addition of HT and the reconstitution of functional EBNA3C, Rb was gradually re-phosphorylated and up-regulated (Figure 4B); concomitantly, expression of p130 decreased as cells entered the proliferation cycle, and p107 expression increased.

### Regulation of H3K27me3 and H3K4me3 across the *p16^INK4A^* locus in LCL 3CHT requires EBNA3C

The repression of *p16^INK4A^* by PcG silencing complexes adding H3K27me3 marks to chromatin has been well characterized in primary fibroblasts. In pre-senescent proliferating fibroblasts the H3K27me3 mark forms a broad peak centred on the first exon of *p16^INK4A^*; induction of senescence is associated with displacement of PcG silencers and a profound reduction of H3K27me3 on the chromatin associated with *p16^INK4A^* exon 1 [40], [44], [45], [46].

**Figure 5:**
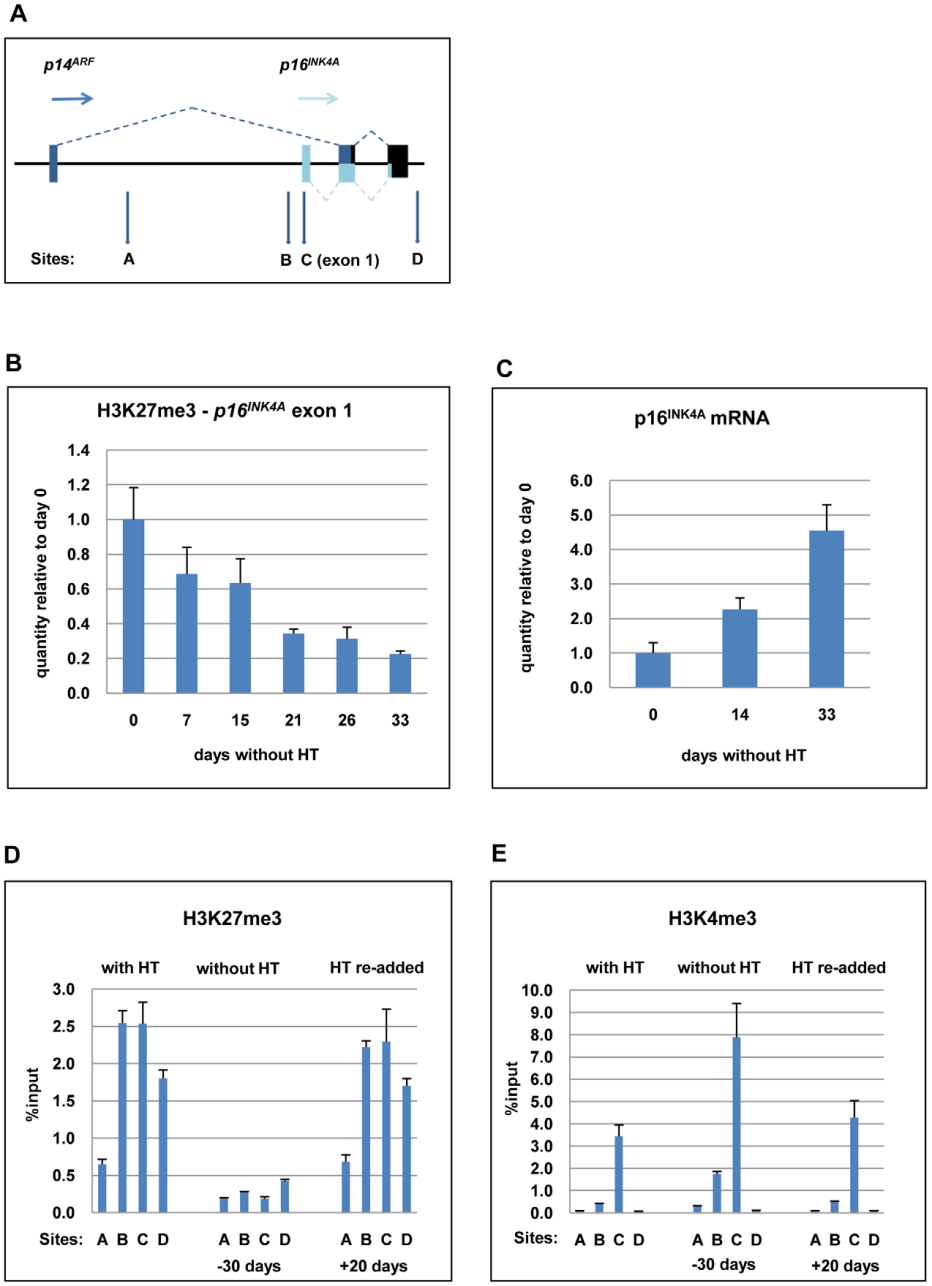
ChIP analysis to quantify the H3K27me3 and H3K4me3 marks on *p16^INK4A^* exon 1 when EBNA3C is inactivated and re-activated in LCL 3CHT. (**A**) Schematic of the human *p16^INK4A^-ARF* locus showing the location of coding exons (boxes) and transcription start sites (horizontal arrows) – not drawn to scale. The vertical (A-D) arrows refer to the approximate locations of primer pairs used for qPCR analysis of precipitated chromatin (as described in Materials and Methods). (**B**) ChIP analysis of H3K27me3 distribution on exon 1 (site C) of *p16^INK4A^*. The histogram shows a decline in H3K27me3 relative to a cycling LCL 3CHT (day 0). (**C**) Corresponding changes in *p16^INK4A^* mRNA quantified by qRT-PCR. (**D**) ChIP analysis of H3K27me3 distribution across the *p16^INK4A^-ARF* locus in LCL 3CHT proliferating in the presence of HT, after 30 days without HT and 20 days after re-adding HT. (**E**) ChIP analysis of H3K4me3 the *p16^INK4A^-ARF* locus in LCL 3CHT proliferating in the presence of HT, after 30 days without HT and 20 days after re-adding HT.

We hypothesised that the up-regulation of *p16^INK4A^* transcription in the absence of functional EBNA3C might result from loss of PcG-mediated repression. Therefore ChIP analyses were performed to assess the level of H3K27me3 in the *p16^INK4A^* locus (see schematic in Figure 5A) in LCL 3CHT cultured with and without HT. Upon EBNA3C inactivation by the removal of HT, H3K27me3 gradually decreased at the *p16^INK4A^* exon 1, as transcription increased (Figure 5B and C). This process could be reversed by reconstitution of functional EBNA3C after re-addition of HT (see below). The time taken for H3K27me3 depletion correlated with the length of time in culture without functional EBNA3C and was consistent with the rate of p16^INK4A^ induction. Equivalent results were obtained when similar ChIP experiments were performed on an independent LCL 3CHT and using two different sets of *p16^INK4A^* exon 1-specific primers (Figure S2 and data not shown).

Recently it became clear that the transcriptional status of *p16^INK4A^* is not determined by H3K27 tri-methylation alone, but rather by the interplay between H3K27me3 and H3K4me3 modifications [44]. Therefore ChIP analyses were performed to assess the quantities of both modifications at the *p16^INK4A^* locus in LCL 3CHT cultured with and without HT (Figures 5D and E). EBNA3C inactivation (labelled -30 days) affected epigenetic marks at the *p16^INK4A^* locus in a manner consistent with transcriptional activation; the repressive H3K27me3 mark was reduced while the activation-related H3K4me3 increased. After EBNA3C reactivation (labelled +20 days), the epigenetic modifications at *p16^INK4A^* locus were apparently reversed.

Further ChIP assays were used to confirm the specificity of *p16^INK4A^* regulation. The quantities of H3K27me3 and H3K4me3 at *p16^INK4A^* exon 1 (primer set C – indicated by boxes) were compared to the quantities at various other sites in the *CDKN2A* locus, including a region (site A) located 4.5kb downstream of *p14^ARF^* transcription start site (Figures 5A, D and E). As described previously [45] in human primary fibroblasts H3K27me3 marks are broadly distributed across the *CDKN2A* locus, peaking in exon 1, but extending into the region corresponding to A. Similarly, this region in LCL 3CHT was associated with some degree of H3K27me3 – particularly in cycling cells. However – whether or not EBNA3C is active – H3K4me3 is detected on exon 1 (site C), but at site A it is always completely absent. In parallel, ChIP experiments were performed with an IgG antibody of the same isotype as the anti-H3K27me3 or -H3K4me3 antibodies to assess the level of background and no significant binding was observed at any site (data not shown). The presence of two inversely regulated modifications excludes the possibility that the reduction in histone methylation is due to nucleosome re-positioning away from *p16^INK4A^* resulting in a reduction of the total histone H3 at the locus.

Relatively high quantities of H3K4me3 were detected at the *p16^INK4A^* exon 1 (site C) compared to site A, even when EBNA3C was active (in LCL 3CHT with HT and with HT re-added) and therefore repressing *p16^INK4A^*. This suggests that *p16^INK4A^* exon 1 in LCLs might contain a ‘bivalent’ or ‘poised’ chromatin domain [47].

### EBNA3C-mediated regulation of *p16^INK4A^* does not require Rb

**Figure 6:**
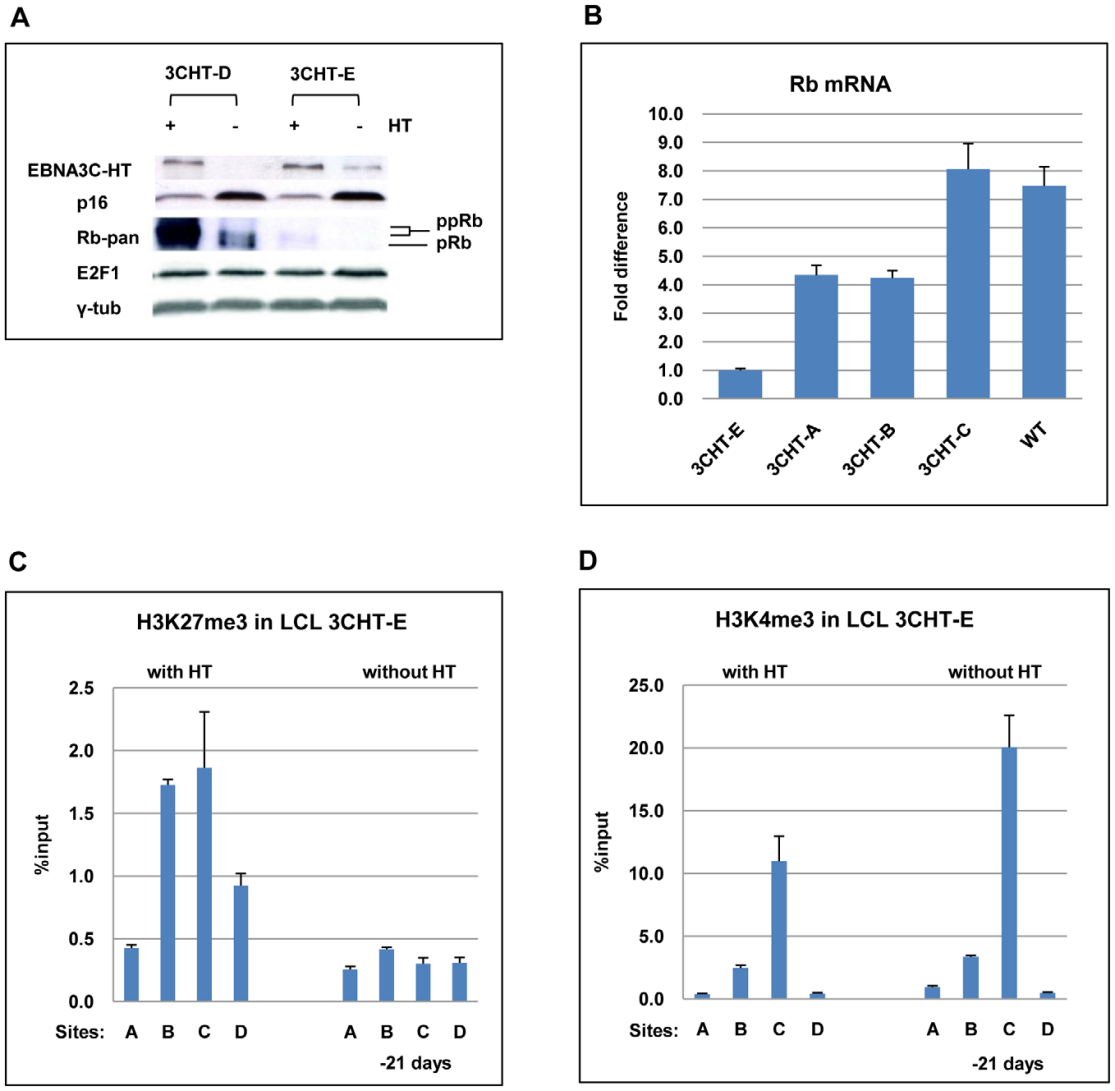
EBNA3C-mediated regulation of *p16^INK4A^* does not require Rb. (**A**) Western blot analysis of whole cell lysates from two LCL 3CHT (-D and -E) cultured with (+) or without (-) HT for 26 days. Although Rb is undetectable in LCL 3CHT-E, when HT is removed from the growth medium, EBNA3C decreases and p16^INK4A^ (p16) increases. E2F1 and γ-tubulin (γ-tub) levels do not alter. (**B**) Steady-state levels of Rb mRNA in LCL 3CHT-E relative to 3 other LCL 3CHT and a WT-BAC LCL all cultured with HT, quantified by qRT-PCR. (**C**) ChIP analysis of H3K27Me3 distribution across the *p16^INK4A^-ARF* locus in LCL 3CHT-E cells (expressing little or no Rb) with HT or without HT in the growth medium. (**D**) ChIP analysis of H3K4me3 distribution across the *p16^INK4^-ARF* locus essentially as described in (C).

To avoid biases due to the genetic background of a single donor, we decided to confirm our findings using newly established LCL 3CHT lines from several different donors. It was soon noted that one of these cell lines did not arrest after the removal of HT from the medium. Western blotting with a pan-specific anti-Rb antibody failed to detect Rb protein in this cell line (LCL 3CHT-E) cultured with or without HT (Figure 6A). Consistent with this, qPCR showed that LCL 3CHT-E expressed low levels of Rb mRNA in comparison to other LCL 3CHT lines (Figure 6B). It is well established that functional Rb can regulate p16^INK4A^ levels through a negative feedback loop and that Rb-negative tumours (such as carcinoma of cervix) and tumour-derived cell lines can express high levels of p16^INK4A^ [23], [48]. However, even in this LCL in which Rb cannot be detected, *p16^INK4A^* was still repressed in the presence of active EBNA3C, and this was relieved after EBNA3C inactivation by the removal of HT (Figure 6A). When ChIP analyses for H3K27Me3 (and H3K4Me3) marks across the *CDKN2A* locus were performed on the LCL expressing no detectable Rb (LCL 3CHT-E) similar patterns to those seen for LCLs expressing Rb protein were seen (compare Figures 6C and D with Figures 5D and E). That is, high levels of H3K27Me3 occupied exon 1 (site C) in the presence HT, while there were low levels of this repressive mark in its absence. The reverse was true for H3K4me3. This reinforces our view that the epigenetic regulation of *p16^INK4A^* by EBNA3C is independent of Rb expression.

### EBNA3A also contributes to the regulation of *p16^INK4A^*

Recently EBNA3A was shown to regulate *p16^INK4A^* in a microarray study using EBNA3A-knockout (KO) LCLs [8]. The efficiency with which LCLs can be established using EBNA3A-KO is lower than with wild type virus, but by infecting peripheral blood leukocytes (PBLs) including macrophages that transiently act as feeder cells, we generated two independent EBNA3A-KO LCLs.

**Figure 7:**
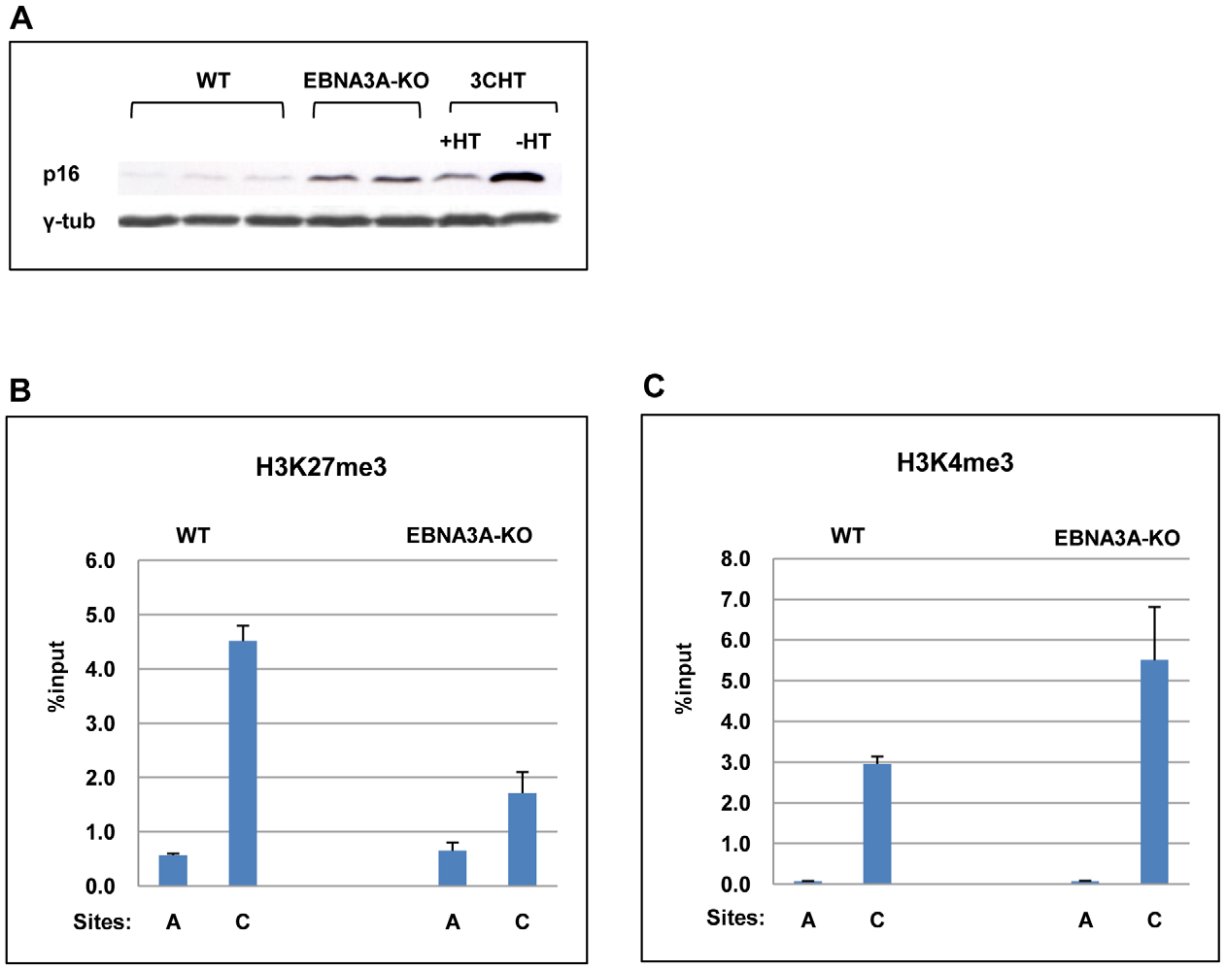
EBNA3A contributes to the regulation of *p16^INK4A^*. (**A**) Western blot analysis of three LCLs established with virus derived from B95.8-EBV BAC (WT) and two established with EBNA3A-KO recombinants. The steady state levels of p16^INK4A^ (p16) are elevated in the EBNA3A-KO cells relative to the WT-EBV infected cells. It should be noted that similar results were reported using a larger panel of EBNA3A-KO LCLs [8]. An LCL 3CHT (3CHT) with (+) or without (-) HT is shown for comparison. (**B**) **&** (**C**) ChIP analysis of H3K27me3 and H3K4me3 distribution on exon 1 (site C) and site A in the *p16^INK4A^-ARF* locus from an EBNA3A-KO LCL (3AKO) and a WT-EBV LCL (WT).

Since EBNA3A and EBNA3C co-operate to epigenetically regulate the cellular gene *BIM* in BL31 cells, we wanted to ask whether EBNA3A might also cooperate with EBNA3C in modifying chromatin at the *p16^INK4A^* locus. The two EBNA3A-KO LCL were validated by probing western blots of protein extracts for the major latent EBV proteins; as previously described for these knockouts in a BL background there were no consistent differences in EBV gene expression ([37]; data not shown). Although only two independent EBNA3A-KO lines were investigated, further characterization showed that – consistent with the report from Hertle and colleagues [8] – p16^INK4A^ protein expression is elevated in both EBNA3A-KO LCLs relative to WT (B95.8)-BAC infected LCL (Figure 7A).

A comparison of steady state levels of H3K27me3 and H3K4me3 at the *p16^INK4A^* locus revealed that in the absence of EBNA3A, the ratio of H3K27me3 to H3K4me3 associated with exon 1 was reversed relative to LCLs established with WT EBV BACs (Figures 7B and C). The low level of H3K27me3 and high level of H3K4me3 are consistent with a more transcriptionally active locus and the higher levels of p16^INK4A^ protein detected in the EBNA3A-knockout lines. This suggests that EBNA3A, together with EBNA3C, is involved in the chromatin remodelling of *p16^INK4A^.*

In the EBNA3A-KO LCLs used for microarray analysis, the level of Rb transcripts was reported to be lower than in LCLs carrying WT EBV [8]. The two EBNA3A-KO LCLs described here expressed similar levels of Rb to the WT EBV infected cells. However, there was substantially more of the hypophosphorylated form when EBNA3A was not expressed (data not shown).

### LCLs expressing CtBP-binding mutants of EBNA3A and EBNA3C grow relatively poorly and express higher than normal levels of p16^INK4A^

We showed previously that EBNA3A and EBNA3C mutants that are unable to bind CtBP are severely impaired in their ability to transform primary rat embryo fibroblasts in co-operation with activated Ha-RAS [16], [17]. Since this assay, in part, measures the ability of proteins to overcome p16^INK4A^-mediated premature senescence and because CtBP is involved in the repression of *p16^INK4A^* in primary human fibroblasts and keratinocytes [22], it seemed appropriate to ask whether the interaction of CtBP with EBNAs 3A and C is necessary for the modulation of p16^INK4A^ expression in LCLs.

**Figure 8:**
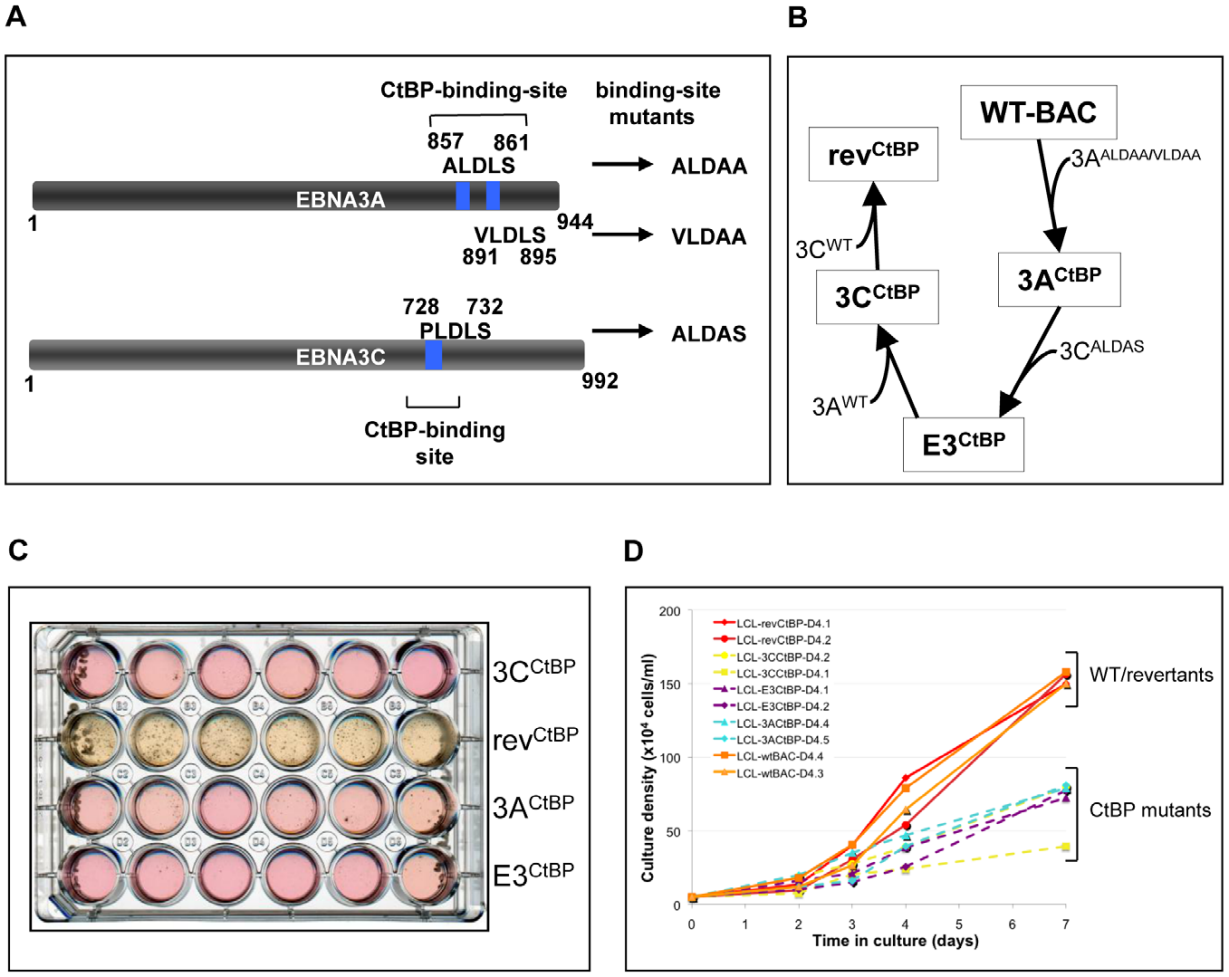
LCLs expressing CtBP-binding mutants of EBNA3A and EBNA3C grow relatively poorly. (**A**) Schematic showing mutations of CtBP-binding sites in EBNA3A and EBNA3C that were introduced into the CtBPM recombinant viruses. EBNA3C includes a single consensus PLDLS site that was mutated to ALDAS [16]. EBNA3A includes two non-canonical CtBP-binding sites that synergise to produce very efficient binding to CtBP; the ALDLS site was mutated to ALDAA and the VLDLS site was mutated to VLDAA [17]. (**B**) Schematic showing the generation of a series of CtBP-binding mutant EBVs, by serially mutating the EBNA3 CtBP binding sites, and then reverting them, creating both single and double CtBP mutant EBVs, and a revertant. (**C**) A plate of cells 40 days after infection of PBLs with 1000 rgu of virus per well. Difference in the colour of the medium and the visible density/size of cell clumps in the wells are clear indicators that CtBP-mutant LCLs grow out more slowly than wild-type (not shown) and revertant LCLs. This appears to be more striking in 3C^CtBP^ and E3^CtBP^ LCLs. (**D**) Established LCLs (approximately 3 months post-infection) were seeded at 5×10^4^ cells/ml and counted each day for a week. CtBP-mutants (dashed lines) grow more slowly, and/or have a lower maximum cell density than wild-type and revertant LCLs (solid lines). Two cell lines were tested for each mutant.

Mutations in EBNA3A and EBNA3C that completely ablate their capacity to bind CtBP have been described ([16], [17]; schematic in Figure 8A). These mutations were serially engineered into the B95.8 EBV-BAC creating CtBP-binding mutants of EBNA3A and EBNA3C, both individually and together, along with revertant virus (Figure 8B). These viruses were used to establish LCLs from PBL and fully validated by mutation-specific PCR, CtBP IP and western blotting for EBV latent proteins (Figure S3). Although latent EBV gene expression generally appeared unaffected by these point mutations it was soon noticeable that population growth was impaired. Limiting dilution analysis of infected PBL was performed in an attempt to quantify this impairment. In terms of the number of wells that contained clumps of cells characteristic of LCL outgrowth after 40 days, there was no substantial difference between the immortalization efficiency of the wild-type and revertant viruses, as compared to the CtBP mutants (data not shown). However, it was clear that the rate of outgrowth of the CtBP mutant LCLs was considerably slower than the wild-type LCLs. This effect is strikingly illustrated by the image of the plate in which the LCLs were grown (Figure 8C). Both the colour of the culture medium and the visible cell clumps in the wells show the much more rapid growth of the revertant LCLs as compared with the mutants. Also notable was the tendency of the mutant EBVs to sometimes grow as a single large clump of cells in the presence of a large number of smaller clumps (Figure 8C, eg wells A4 and C5). This may indicate natural selection driving phenotypic changes in the cells to allow the more robust clones to emerge as LCLs. Even once LCLs are established, CtBP-mutant LCLs continue to show a growth defect, exhibiting reduced rate of population growth relative to wild-type and revertant LCLs (Figure 8D). They also tended to have a much lower maximum cell density, with CtBP-mutant LCLs struggling to grow much beyond 0.7×10^6^ cells/ml.

**Figure 9:**
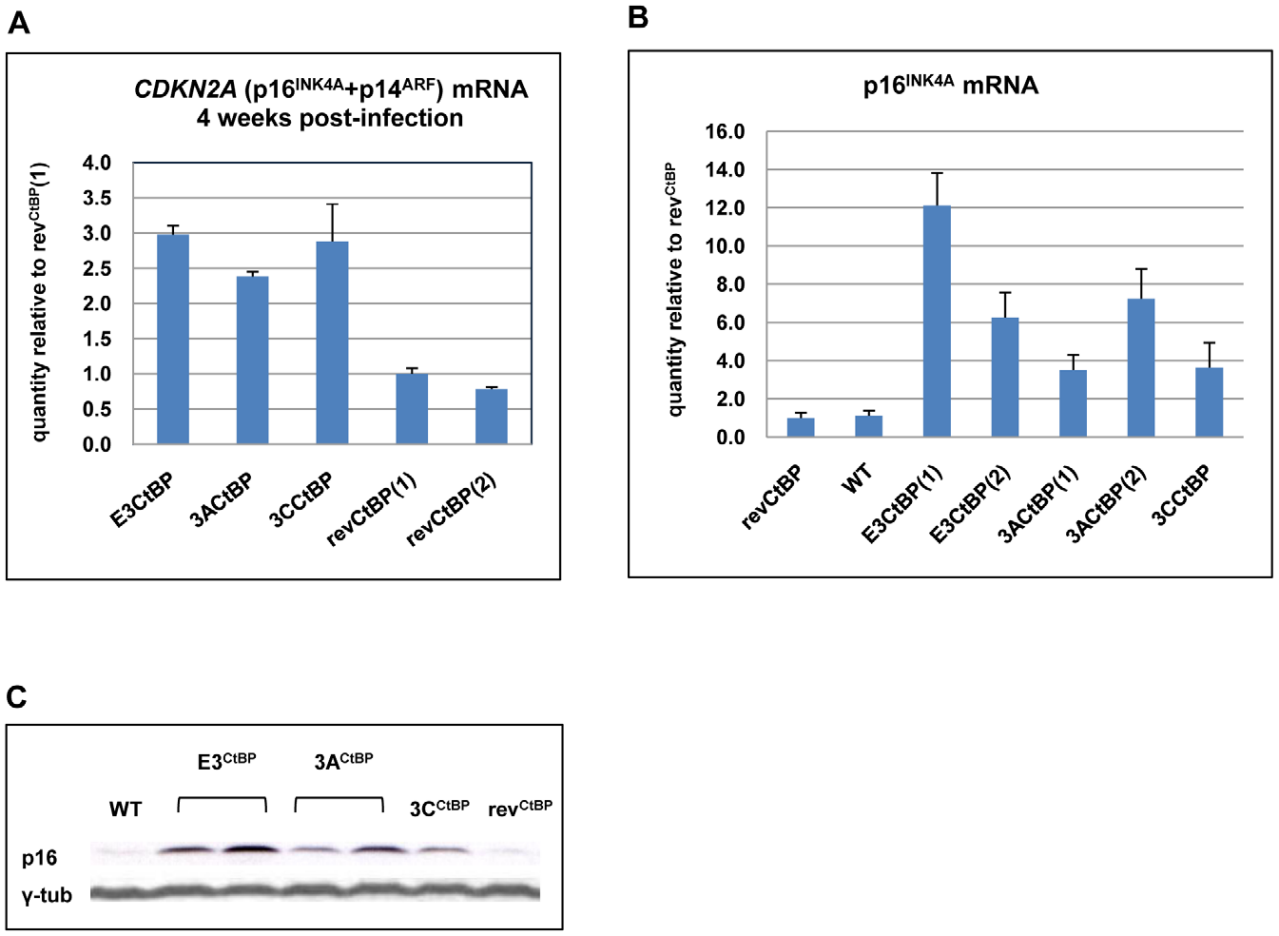
Expression of p16^INK4A^ is increased in CtBPM LCLs relative to revertant and WT LCLs. (**A**) E3^CtBP^, 3A^CtBP^, 3C^CtBP^ and two rev^CtBP^ LCLs were harvested 28 days after the infection of primary B cells with the recombinant EBVs. RNA was extracted and the relative levels of *CDKN2A* transcripts were quantified by qRT-PCR. (**B**) RNA was extracted from two established E3^CtBP^ LCLs, two 3A^CtBP^ LCLs, 3C^CtBP^ LCL, a revertant (rev^CtBP^) and a WT-EBV LCL. The relative levels of *p16^INK4A^* transcripts were quantified by qRT-PCR. (**C**) Western blot analysis of protein extracts from two E3^CtBP^ LCLs, two 3A^CtBP^ LCLs and a 3C^CtBP^ LCL all established from a single donor. A revertant (rev^CtBP^) and a WT-EBV LCL are shown for comparison. Levels of p16^INK4A^ (p16) are shown relative to γ-tubulin (γ-tub).

When p16^INK4A^ transcripts and protein were quantified by qRT-PCR and western blotting respectively (Figures 9A, B and C) it was apparent that all the mutants express more p16^INK4A^ mRNA and protein than either the WT-BAC or revertant-LCL cells. We assume that this increase in p16^INK4A^ contributes to the impaired outgrowth of the mutant-carrying LCLs.

### Interaction of EBNA3A and EBNA3C with CtBP is necessary for the chromatin remodelling associated with the repression of *p16^INK4A^*

**Figure 10:**
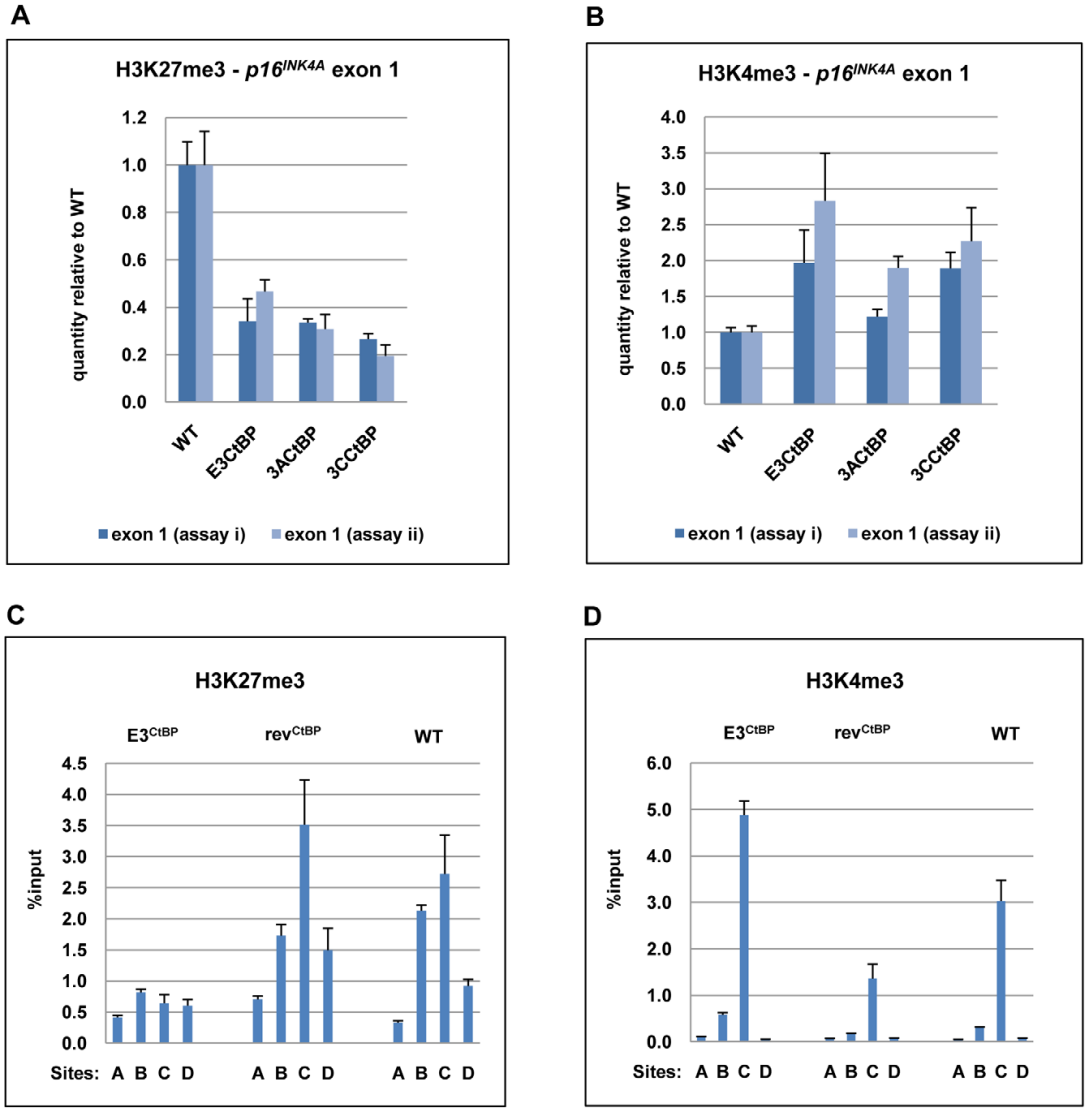
The interaction of EBNA3A and EBNA3C with CtBP is necessary for the chromatin remodelling associated with the repression of *p16^INK4A^.* (**A**) & (**B**) ChIP analysis of H3K27me3 and H3K4me3 in *p16^INK4A^* exon 1 (site C) performed in duplicate (assays i & ii) on an E3^CtBP^ LCL, a 3A^CtBP^ and a 3C^CtBP^. Similar analysis of a WT-EBV LCL is shown for comparison. (**C**) & (**D**) ChIP anaysis of H3K27me3 and H4Kme3 distribution across the *p16^INK4A^-ARF* locus in an E3^CtBP^ LCL. Similar assays performed on revertant (rev^CtBP^) and WT-EBV LCLs are shown for comparison. The sites correspond to those shown in Figure 5A.

CtBP has been implicated in PcG-mediated repression [19], [49], [50], [51] and may be directly involved in the chromatin remodelling of *p16^INK4A^* [22]. ChIP analysis within *p16^INK4A^* exon 1 showed that all the CtBP-mutant LCLs exhibited a significant reduction of the H3K27Me3 mark relative to a WT-EBV LCL. There was generally a corresponding increase in the level of the activation mark H3K4me3 relative to WT (Figure 10A). A detailed comparison of a WT-BAC LCL and a revertant LCL (rev^CtBP^) with a double CtBP-mutant LCL (E3^CtBP^) across the *p16^INK4A^* locus was also performed. The double CtBPM mutant profile closely resembled those of LCL 3CHT cells grown without HT and therefore lacking a functional EBNA3C. In contrast the WT and revertant profiles – as would be expected – resembled LCL 3CHT cultured in the presence of HT (compare Figures 10B and C with Figures 5D and E). We conclude that the interaction of both EBNA3A and EBNA3C with CtBP is important for EBV-mediated chromatin remodelling and repression of *p16^INK4A^* during B cell transformation. This may explain why these protein:protein interactions are particularly important for the efficient outgrowth and establishment of LCLs.

Although the E3^CtBP^ LCL examined here appears to have an active *p16^INK4A^* locus and relatively high levels of p16^INK4A^ protein, the cells are clearly capable of cell division – leading us to suspect that some other element of the Rb-axis might be compromised. An examination of these cells, and other CtBP-mutant cells, showed that proliferation was probably not completely inhibited because in both E3^CtBP^ and 3A^CtBP^ LCLs expression of Rb is often reduced (see for example Figure S4). Since there will be considerable selection pressure during the establishment of an LCL, this is probably the result of unidentified compensatory lesions occurring in the Rb-axis early after infection and the outgrowth of the more robustly proliferating clones.

## Discussion

Here we show that EBNA3A, EBNA3C and CtBP are all involved in the epigenetic repression of p*16^INK4A^* expression that is necessary for the proliferation of EBV-transformed B cells.

The *CDKN2A* locus in the human genome encodes two tumour suppressor proteins – p16^INK4A^ and p14^ARF^ – that are encoded by transcripts consisting of a unique first exon and shared second and third exons (Figure 5A). However, the proteins are structurally unrelated since p14^ARF^ utilizes an alternative reading frame to that used for p16^INK4A^ [23]. The *p16^INK4A^* promoter region is epigenetically regulated by the interplay of repressive modifications, eg H3K27me3, and activating trimethylation of lysine 4 at histone H3 (H3K4me3) in human stem cells, primary fibroblasts and cancer cells [44], [45], [52]. In stem cells H3K27me3 and H3K4me3 modifications co-localize at the *p16^INK4A^* promoter and form what is known as a ‘bivalent’ chromatin domain. Such domains are thought to repress or silence genes while keeping them poised for activation [47], [53]. This epigenetic repression of the *CDKN2A* locus is mediated by polycomb group proteins (PcG) which are thought to silence or repress target genes through the formation of higher order chromatin structures (eg chromatin looping) and by ubiquitinylating H2A at K119 – a histone modification that interferes with the binding of RNA Polymerase II and transcriptional elongation [39], [54]. The H3K27me3 demethylase JMJD3 contributes to plasticity of the *p16^INK4A^* locus, and functions in a switch from the repressive chromatin in response to oncogenic stress [45], [46]

The data presented here have shown that compromising EBNA3C function initiates chromatin remodelling that resets the epigenetic status of *p16^INK4A^* locus. In the presence of active EBNA3C, the *p16^INK4A^* locus displays repressive chromatin and the locus is closed for transcription, but when EBNA3C is inactivated the chromatin is remodelled into an active state that supports transcription. These changes in epigenetic modifications of the *p16^INK4A^* locus might be a consequence of EBNA3C acting on the *p16^INK4A^* locus either directly or indirectly. Results of experiments performed with EBNA3A-KO LCLs and CtBP-binding mutants of both EBNA3A and 3C are all consistent with EBNA3A cooperating with EBNA3C, and both interacting with CtBP in the regulation of these epigenetic chromatin modifications of the *p16^INK4A^* locus.

### Potential mechanisms

Although at least one virus – *Paramecium bursaria* Chlorella virus 1 – encodes its own histone methyltransferase that catalyses the methylation of histone H3K27 and represses a multitude of host genes [55], this is unlikely to be the case of EBNA3C or EBNA3A. Even though the crystal structure of neither is available and their secondary structure is difficult to predict [5], sequence homology studies fail to identify a potential methyltransferase domain in either EBNA3A or EBNA3C.

The consensus of opinion is that the regulation of *p16^INK4A^* is primarily under the control of members of the polycomb group of proteins (PcG). As already indicated, these multi-component repressor complexes generate histone modifications – including H3K27me3 – that are characteristic of silent chromatin. These marks are heritable and may affect the whole *CDKN2A* locus [23]. In *Drosophila*, repression by PcG complexes spreads from polycomb response elements (PREs) located within a genomic regulatory locus, but the equivalent of PREs have not been identified in mammalian cells. The mechanism of targeting PcGs specifically to the *p16^INK4A^* locus is unknown and, more generally, how the DNA-binding specificity of PcG complexes is achieved remains a key question in mammalian biology. The most likely candidates for PcG recruiting factors are sequence-specific transcription factors or long non-coding RNAs (nc-RNAs) [40], so a major challenge for the immediate future is to establish whether EBNAs 3A and 3C together deregulate or interact with sequence specific transcription factors or nc-RNAs that normally regulate *p16^INK4A^ and/or BIM*.

No consistent changes in the expression of PcG proteins or the H3K27me3-specific demethylase JMJD3 have been identified in LCLs expressing inactivated or mutated EBNAs 3A or 3C ([8]; our unpublished observations). Furthermore, although it has been shown that LMP1 is a negative regulator of *p16^INK4A^* in fibroblasts and epithelial cells [56], [57], LMP1 expression was not reduced in any of the mutant LCLs studied here or reported elsewhere (Figures S1 and S3; [8], [35]. We therefore do not think LMP1 signalling plays a significant role in the repression of *p16^INK4A^* in B cells.

### Consequences of epigenetic repression in lymphomagenesis

Epigenetic changes are by definition heritable, but not always irreversible. Certain loci exhibit a high degree of plasticity and are poised for rapid activation. Examples include families of developmental genes such as those in the *HOX* locus and tumour suppressor genes that become active when aberrant pro-proliferative signals are detected [23], [40]. Our data suggest that the repression of *p16^INK4A^* locus by EBNA3C (presumably cooperating with EBNA3A which is constitutively expressed) in the LCL 3CHT system is reversible. H3K27me3 is known to cause the local formation of heterochromatin that is labile or readily reversible, but this type of histone methylation can also facilitate methylation of DNA of the same region, particularly in the development of cancer. DNA methylation represents a more stable modification and can ‘fix’ the repression of the locus. It has been shown that promoter regions bound by PcG which remain largely unmethylated on CpG dinucleotides in normal tissues, serve during tumorigenesis as a map to direct DNA methylation. CpG islands methylated *de novo* in cancer have often been previously marked by the presence of PcG proteins and H3K27me3 [58], [59], [60], [61], [62], [63]. We have shown previously that this is the case for *BIM* in EBV-positive lymphomas [38]. It is therefore also likely that during EBV-mediated lymphomagenesis *p16^INK4A^* is repressed as a result of EBNA3A/C-induced histone modifications that then act as a focus for DNA methyltransferases to initiate the more stable cancer-associated CpG methylation. This is certainly consistent with the limited data on EBV-positive BL, that suggest the *p16^INK4A^* locus is nearly always silenced by DNA methylation of exon 1 in tumour-derived cell lines and in many BL biopsies [64], [65].

### Limitations of the conditional EBNA3C-HT system

EBNA3C is absolutely essential for the transformation of primary B cells by EBV, therefore the successful establishment of LCL 3CHT reassures us that EBNA3C modified by a C-terminal fusion retains most of the functions required for transformation. However, there appear to be at least two limitations of the EBNA3C-HT fusion system. Firstly, even in the presence of HT the repression of *p16^INK4A^* is not complete. The amount of p16^INK4A^ is higher than in WT-EBV LCL and is almost equivalent to the accumulation seen in EBNA3A-KO and CtBP-mutant LCLs (Figures 7A and 9C). The elevated levels of p16^INK4A^ in LCL 3CHT, even in the presence of HT might indicate that EBNA3C activation is compromised in a subpopulation of cells and these cells exit from the cell cycle. Alternatively, and perhaps more likely, fusion of EBNA3C with the large estrogen receptor domain might partially inhibit EBNA3C function in all cells in the population, even in the presence of HT.

A second limitation to the utility of these cells for studying EBNA3C function is the considerable delay between removing or re-adding HT from/to the culture medium and the change in levels of p16^INK4A^. The first signs of p16^INK4A^ increase are seen about 10 days after removing HT from the medium and the first signs of *p16^INK4A^* repression are apparent no sooner than two days after re-addition of HT. This may be caused by the EBNA3C-HT fusion not being quite equivalent to WT EBNA3C (as discussed above) or could indicate that *p16^INK4A^* is not a direct target of EBNA3C. A similar phenomenon occurs during the induction of p16^INK4A^ by activated RAS. It takes approximately 5 days to fully activate *p16^INK4A^* by RAS. Since such an extended timeframe is not compatible with the signalling dynamics of the RAF-MEK-ERK cascade, it has been suggested that mutant RAS leads to the accumulation of intracellular stresses and subsequent activation of p38 [23]. It is possible that EBV latent gene expression minus EBNA3C or EBNA3A sets up some sort of dis-equilibrium that results in intracellular stress, but preliminary data suggest that this is not mediated by p38 activation (data not shown). Alternatively, it has been suggested that the slow kinetics of *p16^INK4A^* activation might reflect the need to displace either repressive histone or repressive PcG-mediated DNA methylation [23], [60], [66] and this could be the case in B cells latently infected with EBV.

### The role of CtBP

The role of CtBP in EBNA3A/3C-mediated repression of *p16^INK4A^* is intriguing but not yet understood. CtBP was discovered because of its binding to adenoviral oncoprotein E1A. Binding of E1A to CtBP antagonizes the function of CtBP, and E1A mutants unable to bind CtBP show enhanced efficiency of transformation [67]. In contrast, EBNA3A and EBNA3C mutants unable to bind CtBP were less effective in transforming and immortalizing primary rat embryo fibroblasts in cooperation with Ha-RAS [16], [17]. Furthermore we showed that Marek's disease virus, a herpesvirus that induces T cell lymphoma in chickens, requires its nuclear oncoprotein MEQ to bind CtBP for tumorigenesis [68]. Consistent with the role of CtBP in rodent cell transformation by EBNA3A and EBNA3C, LCLs produced by immortalization with CtBP-binding mutant viruses grow out much more slowly than WT-EBV LCLs and fail to effectively repress *p16^INK4A^*. Taken together the data suggest that binding of EBNA3A and EBNA3C to CtBP augments transformation efficiency and LCL outgrowth by aiding the establishment or maintenance of the H3K27me3 mark on *p16^INK4A^* exon 1. CtBP-containing complexes have been previously linked to chromatin remodelling including demethylation of H3K4, and CtBP has been shown to recruit PcG silencers to certain genes in mammalian cells [19], [50]. Although both EBNA3A and EBNA3C can be immunoprecipitated from LCLs with CtBP [16], [17] and under standard conditions EBNA3A and EBNA3C can be reciprocally co-precipitated (our unpublished data) we have been unable to ChIP EBNA3A/3C-CtBP complexes on either the *p16^INK4A^* or *BIM* promoters (data not shown). We do not know whether this is because of technical limitations of the reagents that are available or whether these promoters are not actually direct targets of the complexes. It may be necessary to develop new regents to address this issue. Although we do not yet understand this requirement for CtBP-binding, the data are consistent with recent reports that the C-terminus of EBNA3C – and specifically the PLDLS CtBP-binding site – is necessary to completely rescue proliferation in Akata-derived E3C-HT LCLs cultured without HT [69], [70].

### Selection for the loss of Rb

Serendipitously an LCL 3CHT cell line with no detectable Rb protein was produced. In this cell line, *p16^INK4A^* was repressed in the presence of functional EBNA3C and de-repressed after EBNA3C inactivation. We therefore assume that regulation of *p16^INK4A^* by EBNA3C in LCL 3CHT is an Rb-independent phenomenon. Repression of *p16^INK4A^* in the Rb-negative LCL 3CHT line is unlikely to be functionally relevant, because the main target of p16^INK4A^ is absent. Since EBNA3C repressed *p16^INK4A^* even when no additional proliferation advantage was to be gained, this implies that repression of *p16^INK4A^* is a specific consequence of EBNA3C working in collaboration with EBNA3A and CtBP. Decreased expression of Rb protein was also seen in E3^CtBP^ and 3A^CtBP^ LCLs, (Figure S4) and decreased Rb mRNA has been reported in EBNA3A-KO LCLs [8]. Since the absence of Rb will confer a common proliferative advantage, it is probable that elevated p16^INK4A^ expression in these various cell lines creates a strong selection pressure for the loss, or reduced expression of Rb during transformation.

In summary, we have described a novel mechanism used by EBV to overcome stress-induced growth arrest by preventing the induction of p16^INK4A^ and, by a similar mechanism, enhance cell survival by preventing the induction of pro-apoptotic BIM [37], [38]. To our knowledge this ability to epigenetically inactivate a crucial cell cycle inhibitor and a potent inducer of cell death makes EBV unique among the known DNA ‘tumour viruses’ [71]. Understanding the precise molecular details of EBV-mediated chromatin remodelling of *p16^INK4A^* and the role that CtBP plays in this process should cast new light on the nature of viral oncogenesis and the mysteries of polycomb-mediated gene repression.

## Supporting Information

Table S1 Gene expression qRT-PCR primer sequences.
(0.03 MB DOC)

Table S2 ChIP qPCR primer sequences.
(0.03 MB DOC)

Table S3 Sequence of PCR primers used to validate CtBP mutants.
(0.03 MB DOC)

Figure S1 Validation of LCL 3CHT. Western blot analysis of latency-associated EBV proteins in representative LCL 3CHT-A and -B cultured in the medium with HT and 26 days without HT. LCL 3CHT-E line, with reduced Rb protein expression is included in the panel for comparison.
(0.48 MB TIF)

Figure S2 Changes in H3K27me3 at p16^INK4A^ exon 1 in response to activation of EBNA3C. Histogram shows the decline in H3K27me3 in LCL 3CHT-A cultured for 35 days without HT and H3K27me3 restoration after HT was re-added for 16 days. For details see Figure 5 and Materials and Methods of main manuscript.
(0.21 MB TIF)

Figure S3 Validation of CtBP mutant LCLs. **(A)** EBNA3s in CtBP-mutant viruses fail to bind CtBP. Proteins were immunoprecipitated (essentially as described in [16]) from 500 µg of protein extracted from BL31 cells stably infected by CtBP-mutant or -revertant EBVs using 10 µg of a polyclonal anti-CtBP antibody (C) and an isotype-matched control antibody (Ig). Immunoprecipitates and 5% of the input protein (i) were split between two gels for western blotting with anti-EBNA3A and anti-EBNA3C. As is apparent, the CtBP-binding mutant EBNA3s are not immunoprecipitated by the anti-CtBP antibody, while the wild-type protein is. The similar efficiency of the immunoprecipitation in all cell lines was confirmed by re-probing with the anti-CtBP antibody. **(B)** PCR validation of CtBP mutant LCLs. PCRs specific to either the wild-type or mutant XLDLS motif were performed for 30 cycles, and were positive for DNA from the appropriate cell lines only. LCLs established from donor D1 are shown here. Primer sequences are shown in Table S3. **(C)** Western blot analysis of EBV latent proteins in CtBP-mutant LCLs. This shows that there are no substantive differences in EBV gene expression levels between mutant and wild-type LCLs in two genetic backgrounds. Re-probing the western blots with anti-γ-tubulin antibody confirmed equal gel loading (data not shown). The only consistent change is in the CtBP revertants, which appear to have higher levels of EBNA-LP. There is a possible tendency towards marginally higher levels of LMP1, in the CtBP mutants, but this is not consistently observed in all CtBP mutants, nor in all western blots on the same sample (data not shown).
(1.07 MB TIF)

Figure S4 **(A)** Western blot analysis showing the reduction of Rb protein expression in the established E3CtBP and 3ACtBP LCLs in comparison to revCtBP and WT LCLs (several months post-infection and showing elevated p16INK4A levels for comparison). **(B)** Steady state levels of Rb mRNA were quantified by qRT-PCR. For details see Figure 4 in main text.
(0.29 MB TIF)
