# Supplementary material for: Epigenetic Repression of p16INK4A by Latent Epstein-Barr Virus Requires the Interaction of EBNA3A and EBNA3C with CtBP
Source: PLoS Pathog. 2010 Jun 10;6(6):e1000951. doi: 10.1371/journal.ppat.1000951 (PMC2883600; doi:10.1371/journal.ppat.1000951)
Supplement: Table S1 — Gene expression qRT-PCR primer sequences. (0.03 MB DOC) [file ppat.1000951.s001.doc]

**Table S1: Gene expression qRT-PCR primer sequences**

| **Assay** | **Forward primer** | **Reverse Primer** |
| --- | --- | --- |
| CDKN2A | CATAGATGCCGCGGAAGGT | GATGATCTAAGTTTCCCGAGGTTTC |
| Rb | CCAGCACACCCTGCAGAAT | TGCCATACATGGAACACATCATAA |
| p16^INK4A^ | CCCCTTGCCTGGAAAGATAC | AGCCCCTCCTCTTTCTTCCT |
| **RPLP0** | ACTCTGCATTCTCGCTTCCT | GGACTCGTTTGTACCCGTTG |
| **GNB2L1** | GAGTGTGGCCTTCTCCTCTG | GCTTGCAGTTAGCCAGGTTC |
| **ALAS1** | GGATTCGAAACAGCCGAGTG | TGACATCATTGTGGCGGAAG |

p16^INK4A^ assay was developed by Kia, *et al.*, 2008; RPL0 by Christin Down (unpublished data), GNB2L1 by Zhang, Ding and Sandford, 2005.
