# Supplementary material for: Epigenetic Repression of p16INK4A by Latent Epstein-Barr Virus Requires the Interaction of EBNA3A and EBNA3C with CtBP
Source: PLoS Pathog. 2010 Jun 10;6(6):e1000951. doi: 10.1371/journal.ppat.1000951 (PMC2883600; doi:10.1371/journal.ppat.1000951)
Supplement: Table S2 — ChIP qPCR primer sequences. (0.03 MB DOC) [file ppat.1000951.s002.doc]

**Table S2: ChIP qPCR primer sequences**

| **Assay** | **Forward primer** | **Reverse primer** |
| --- | --- | --- |
| A | GGAGCGATGTGATCCGTTATC | TGAAATCCCAATCGTCTTCCAC |
| B | CTCAAAGCGGATAATTCAAGAGC | AAGCCTTAAGAACAGTGCCACAC |
| C (i) | CCCCTTGCCTGGAAAGATAC | AGCCCCTCCTCTTTCTTCCT |
| C (ii) | AGAGGGTCTGCAGCGG | TCGAAGCGCTACCTGATTCC |
| D | TAGGAGGCCCCATTAAGCATAC | TGTAGTTGCCAGGAGTTGGAGG |

Assays A, B, C(ii), D assays were developed by Barradas *et al*., 2009 and assay C(i) by Kia *et al.*, 2008.

Assay A is positioned 4.5kb downstream of p14^ARF^ transcription start site, assay B 1kb upstream p16^INK4A^ promoter, assay C (i) 85bp in 16^INK4A^ exon1, assay C (ii) at the 3’ end of exon1 and assay D 0.8kb downstream 16^INK4A^ exon 3.
