# Supplementary material for: Epigenetic Repression of p16INK4A by Latent Epstein-Barr Virus Requires the Interaction of EBNA3A and EBNA3C with CtBP
Source: PLoS Pathog. 2010 Jun 10;6(6):e1000951. doi: 10.1371/journal.ppat.1000951 (PMC2883600; doi:10.1371/journal.ppat.1000951)
Supplement: Table S3 — Sequence of PCR primers used to validate CtBP mutants. (0.03 MB DOC) [file ppat.1000951.s003.doc]

**Table S3: Sequence of PCR primers used to validate CtBP mutants**

| Detects | Primer Name | WT/mutant-specific primer | Shared primer |
| --- | --- | --- | --- |
| 3C^CtBP^ | 3C-ALDAS-fwd | ACGCTGCGCTGGATGCAAG | 3CforCtBP-rev CAGGGACCCGCATATCCTGGATA |
| 3C WT | 3C-PLDLS-fwd | GGATCCTGACGCTCCTCTGG |
| 3A WT | 3A-ALDLS-rev | GGATTGACAAATCAAGAGC | 3AforCtBP-fwd TGGATGCTTTGGGGTATACACTCC |
| 3A^CtBP^ | 3A-ALDAA-rev | GGATCGCCGCATCAAGAGC |
| 3A WT | 3A-VLDLS-rev | GGATTGACAAATCAAGAACC |
| 3A^CtBP^ | 3A-VLDAA-rev | GGATCGCCGCATCAAGAACC |
