# Supplementary figures and images for: Epigenetic Repression of p16INK4A by Latent Epstein-Barr Virus Requires the Interaction of EBNA3A and EBNA3C with CtBP
Source: PLoS Pathog. 2010 Jun 10;6(6):e1000951. doi: 10.1371/journal.ppat.1000951 (PMC2883600; doi:10.1371/journal.ppat.1000951)

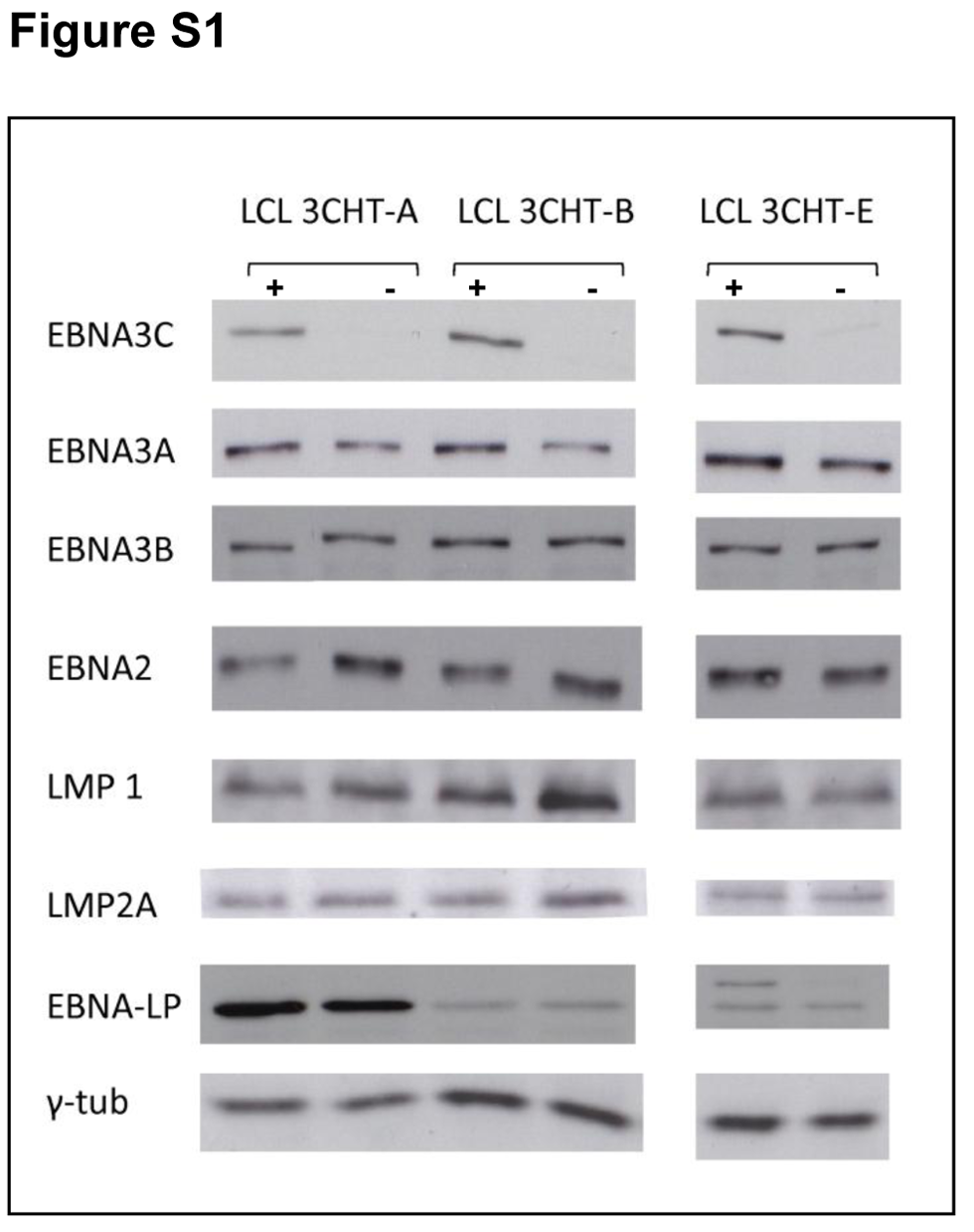

Supplement: Figure S1 — Validation of LCL 3CHT. Western blot analysis of latency-associated EBV proteins in representative LCL 3CHT-A and -B cultured in the medium with HT and 26 days without HT. LCL 3CHT-E line, with reduced Rb protein expression is included in the panel for comparison. (0.48 MB TIF) [file ppat.1000951.s004.tif]

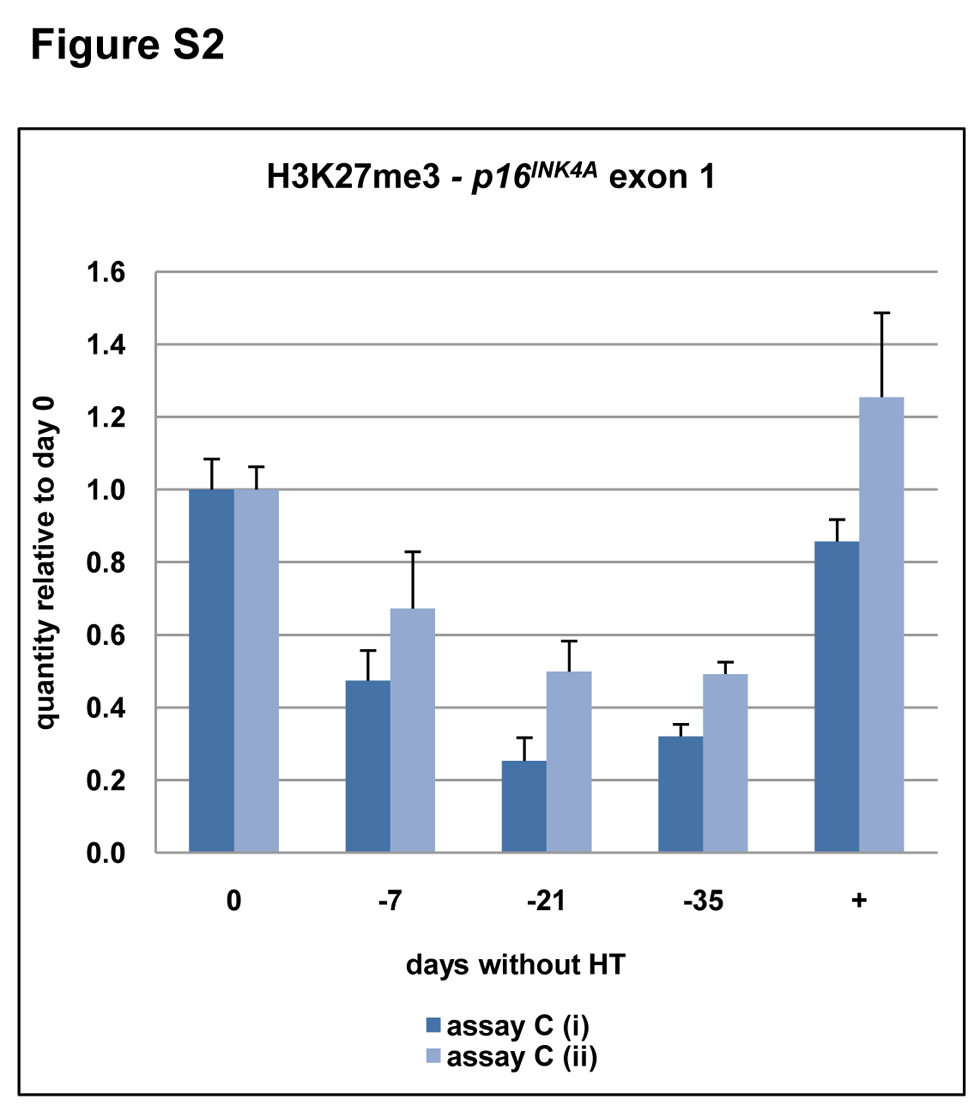

Supplement: Figure S2 — Changes in H3K27me3 at p16INK4A exon 1 in response to activation of EBNA3C. Histogram shows the decline in H3K27me3 in LCL 3CHT-A cultured for 35 days without HT and H3K27me3 restoration after HT was re-added for 16 days. For details see Figure 5 and Materials and Methods of main manuscript. (0.21 MB TIF) [file ppat.1000951.s005.tif]

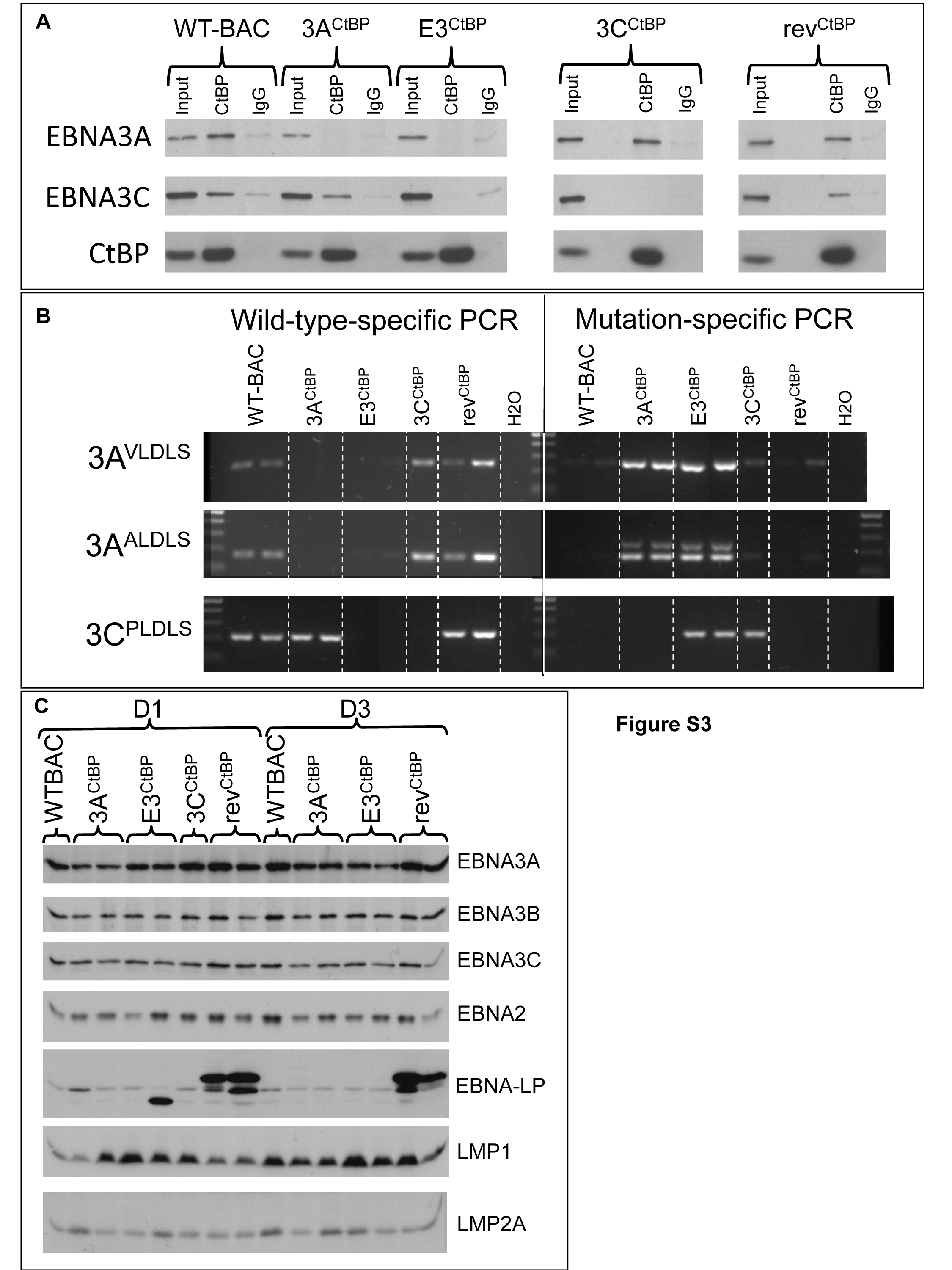

Supplement: Figure S3 — Validation of CtBP mutant LCLs. (A) EBNA3s in CtBP-mutant viruses fail to bind CtBP. Proteins were immunoprecipitated (essentially as described in [16]) from 500 µg of protein extracted from BL31 cells stably infected by CtBP-mutant or -revertant EBVs using 10 µg of a polyclonal anti-CtBP antibody (C) and an isotype-matched control antibody (Ig). Immunoprecipitates and 5% of the input protein (i) were split between two gels for western blotting with anti-EBNA3A and anti-EBNA3C. As is apparent, the CtBP-binding mutant EBNA3s are not immunoprecipitated by the anti-CtBP antibody, while the wild-type protein is. The similar efficiency of the immunoprecipitation in all cell lines was confirmed by re-probing with the anti-CtBP antibody. (B) PCR validation of CtBP mutant LCLs. PCRs specific to either the wild-type or mutant XLDLS motif were performed for 30 cycles, and were positive for DNA from the appropriate cell lines only. LCLs established from donor D1 are shown here. Primer sequences are shown in Table S3. (C) Western blot analysis of EBV latent proteins in CtBP-mutant LCLs. This shows that there are no substantive differences in EBV gene expression levels between mutant and wild-type LCLs in two genetic backgrounds. Re-probing the western blots with anti-γ-tubulin antibody confirmed equal gel loading (data not shown). The only consistent change is in the CtBP revertants, which appear to have higher levels of EBNA-LP. There is a possible tendency towards marginally higher levels of LMP1, in the CtBP mutants, but this is not consistently observed in all CtBP mutants, nor in all western blots on the same sample (data not shown). (1.07 MB TIF) [file ppat.1000951.s006.tif]

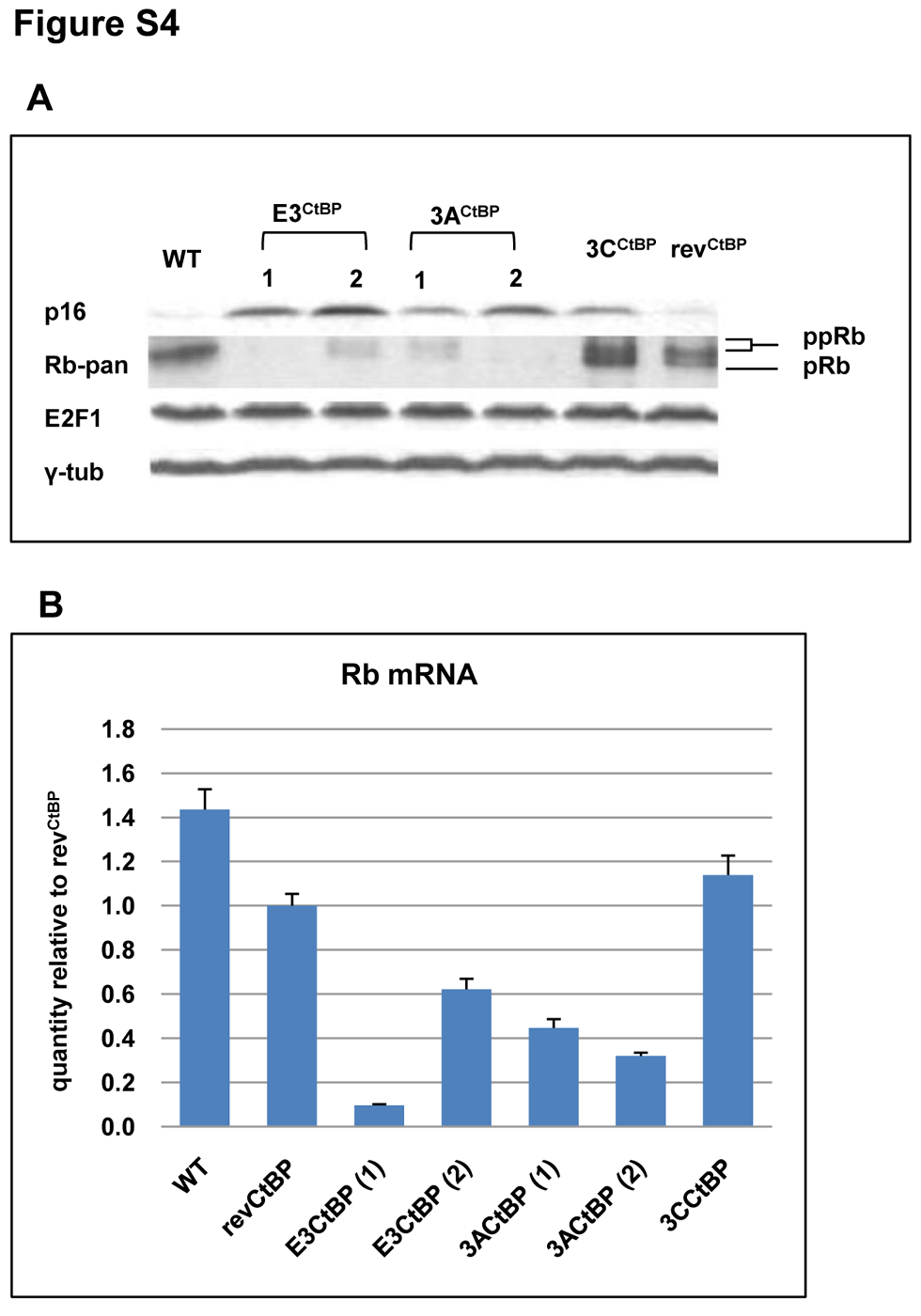

Supplement: Figure S4 — (A) Western blot analysis showing the reduction of Rb protein expression in the established E3CtBP and 3ACtBP LCLs in comparison to revCtBP and WT LCLs (several months post-infection and showing elevated p16INK4A levels for comparison). (B) Steady state levels of Rb mRNA were quantified by qRT-PCR. For details see Figure 4 in main text. (0.29 MB TIF) [file ppat.1000951.s007.tif]
